# A phylogenomic analysis of the role and timing of molecular adaptation in the aquatic transition of cetartiodactyl mammals

**DOI:** 10.1098/rsos.150156

**Published:** 2015-09-30

**Authors:** Georgia Tsagkogeorga, Michael R. McGowen, Kalina T. J. Davies, Simon Jarman, Andrea Polanowski, Mads F. Bertelsen, Stephen J. Rossiter

**Affiliations:** 1 School of Biological and Chemical Sciences, Queen Mary University of London, Mile End Road, London E1 4NS, UK; 2 Australian Antarctic Division, Channel Highway, Kingston, Tasmania 7050, Australia; 3 Center for Zoo and Wild Animal Health, Copenhagen Zoo, Roskildevej 38, Frederiksberg 2000, Denmark

**Keywords:** RNA-sequencing, transcriptome, Cetartiodactyla, mammals

## Abstract

Recent studies have reported multiple cases of molecular adaptation in cetaceans related to their aquatic abilities. However, none of these has included the hippopotamus, precluding an understanding of whether molecular adaptations in cetaceans occurred before or after they split from their semi-aquatic sister taxa. Here, we obtained new transcriptomes from the hippopotamus and humpback whale, and analysed these together with available data from eight other cetaceans. We identified more than 11 000 orthologous genes and compiled a genome-wide dataset of 6845 coding DNA sequences among 23 mammals, to our knowledge the largest phylogenomic dataset to date for cetaceans. We found positive selection in nine genes on the branch leading to the common ancestor of hippopotamus and whales, and 461 genes in cetaceans compared to 64 in hippopotamus. Functional annotation revealed adaptations in diverse processes, including lipid metabolism, hypoxia, muscle and brain function. By combining these findings with data on protein–protein interactions, we found evidence suggesting clustering among gene products relating to nervous and muscular systems in cetaceans. We found little support for shared ancestral adaptations in the two taxa; most molecular adaptations in extant cetaceans occurred after their split with hippopotamids.

## 1. Introduction

Cetaceans are arguably the most specialized of all mammals, having evolved from a terrestrial ancestor to occupy an obligate aquatic niche [1]. Modern cetaceans show numerous phenotypic adaptations for life in the water; aside from the radical reorganization of their forelimbs into fins and loss of hindlimbs, they are able to dive and tolerate low oxygen, and possess modified circulatory and respiratory systems, large brains, hairlessness and transformations in sensory perception [1]. Some cetaceans show extreme longevity, as well as resistance to cancer, wound healing and insulin resistance [2,3]. Other major adaptations pertain to feeding ecology; indeed, modern cetaceans diverged approximately 34 Ma [4,5] into the toothed whales (suborder Odontoceti), which evolved echolocation to hunt using ultrasonic pulses and possess a highly specialized inner ear, and the baleen whales (suborder Mysticeti), which lost their teeth and instead evolved a novel keratinous material for filtering smaller prey [1].

Molecular evidence has revealed that the closest living relatives of the cetaceans are the two extant members of the family Hippopotamidae: the common and pygmy hippopotamus (‘hippo’) (e.g. [6,7]). Members of the Cetacea and Hippopotamidae are grouped together in the monophyletic clade Whippomorpha [8], which in turn is nested within the otherwise terrestrial mammalian order Cetartiodactyla that also includes the even-toed ungulates [9]. Recent molecular evidence suggests that the Whippomorpha diverged from other cetartiodactyls approximately 59 Ma and that the cetaceans and hippopotamids split approximately 55 Ma [5].

Both extant hippo species are adapted for spending long periods of time in water [10]. Like some early cetaceans, they can walk on the bottom of bodies of freshwater due to their thick, dense (pachyosteosclerotic) limb bones [11], and they are predominantly hairless, with thickened, lipid-rich skin that lacks sebaceous glands [12,13]. Hippo skin contains subepidermal capillaries with thickened walls to withstand high blood pressure, an adaptation for heat exchange that has also been reported in highly active species, such as cetaceans [13]. Hippos and cetaceans have some behavioural traits in common such as nursing underwater and subaquatic communication [1,14]. Furthermore, fossil cetaceans and hippos both possess a hyperinflated tegmen tympani of the petrosal bone, which may aid in interpreting the directionality of hearing [15]. Yet despite some shared specializations for life in the water, it is currently unclear to what extent cetaceans and hippos evolved these adaptations independently or whether they are ancestral traits, although some fossil evidence suggests the former [1,10,16]. Indeed, the question of whether the last common ancestor of the Whippomorpha exhibited a terrestrial, semi-aquatic or aquatic lifestyle remains unresolved, and there is particular interest in determining when in their evolutionary history cetaceans gained their specialized traits for living in water [1,10].

Genome-wide scans of selection can offer powerful insights into the evolutionary history of adaptive traits (e.g. [17]). However, all genome-scale studies of molecular adaptation in the Whippomorpha to date have been restricted to a few cetaceans [18–24]. Similarly, inferences of selection associated with the evolutionary transition from land to water have relied heavily on data from toothed whales, used in comparative evolutionary analyses together with the cow (*Bos taurus*) and other more distantly related terrestrial mammals [21,22,24].

To gain a better understanding of the timing and role of natural selection in the transition of cetartiodactyl mammals to a semi-aquatic/aquatic environment, we generated transcriptome data from the common hippopotamus (referred to as ‘hippo’ below) as well as from the humpback whale *Megaptera novaeangliae*. By analysing these together with existing data from two mysticetes and six odonotocetes, we conducted, to our knowledge, the most comprehensive genome-scale dataset of the group to date [18,23–26]. We reasoned that if adaptation to a semi-aquatic environment preceded the split between hippos and whales, then we would expect to see signatures of positive selection in multiple genes linked to an aquatic lifestyle on the ancestral branch of Whippomorpha. If, on the other hand, adaptation to a more aquatic way of life followed the split between these groups, then we might expect independent changes on each lineage. Under both scenarios, we also predicted a greater molecular signature of aquatic adaptation in cetaceans than in hippos, reflecting the more derived body plan in the former.

## 2. Material and methods

### 2.1 Taxon sampling, sequencing and RNA sequencing de novo assembly

New RNA sequencing data for the common hippo *Hippopotamus amphibius* and humpback whale *M. novaeangliae* were generated by pair-end Illumina HiSeq sequencing at BGI (electronic supplementary material, table S1), and combined with published sequence data from genomes or transcriptomes of eight other cetacean species: sperm whale (*Physeter macrocephalus*), Indo-Pacific humpback dolphin (*Sousa chinensis*), minke whale (*Balaenoptera acutorostrata*), fin whale (*Balaenoptera physalus*), finless porpoise (*Neophocaena phocaenoides*), bottlenose dolphin (*Tursiops truncatus*), killer whale (*Orcinus orca*) and Yangtze River dolphin (*Lipotes vexillifer*) [18,23–26].

### 2.2 Orthologue identification and dataset assembly

We obtained orthologous coding DNA sequences (CDSs) across cetaceans and the hippo using reciprocal blastx and tblastn searches, with *T. truncatus* and human as references. Orthologous sequences of 13 other laurasiatherian mammals were obtained from Ensembl [27]. CDS were aligned using PRANK v. 130820 [28] and filtered based on Guidance default parameters [29]. Sequences were further edited and trimmed to avoid problems with missing data and erroneous indels (electronic supplementary material, methods).

### 2.3 Natural selection analyses

**Figure 1.**
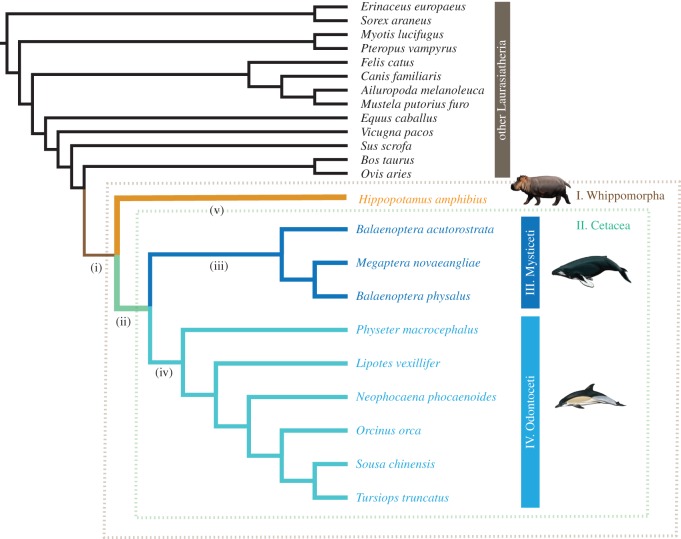
Evolutionary relationships among laurasiatherian mammals as used in molecular evolution analyses. The four clades tested for divergent selection are shown in colour and numbered in uppercase: (I) Whippomorpha (Hippopotamidae + Cetacea); (II) Cetacea; (III) Mysticeti and (IV) Odontoceti. Branches tested for positive selection are numbered in lowercase: (i) Whippomorpha (Hippopotamidae + Cetacea); (ii) Cetacea; (iii) Mysticeti; (iv) Odontoceti and (v) hippo.

To identify episodes of positive selection, we used codon models in codeml of PAML v. 4.4 [30]. We first implemented branch-site model MA to identify sites under selection [30,31] on five focal branches: (i) Whippomorpha (Hippopotamidae + Cetacea); (ii) Cetacea; (iii) Mysticeti; (iv) Odontoceti; and (v) the terminal branch of *H. amphibius* (figure 1). Each branch-site model was compared to a null model using the likelihood ratio test (LRT) with 1 d.f., and sites with Bayes Empirical Bayes posterior probabilities of more than 0.50 were considered significant. To ensure that estimated positive *ω*-values represented genuine selection acting on genes, rather than alignment errors, we filtered out genes in which positively selected sites (PSSs) were found to be highly aggregated within the CDS alignment (median interval distance of PSSs ≤10 codons). The exact numbers of datasets remained in each clade or branch after filtering are given in table 1. Following this step, the associated *p*-values from the LRTs were corrected for multiple testing according to the Benjamini & Hochberg's [32] procedure that corrects the false discovery rate (FDR; *Q*-value) to *Q*<0.10. For further analyses using codon models, see the electronic supplementary material, methods, results and discussion, and table S3.

**Table 1.** Genome-wide analysis for bursts of positive selection.

	initial screen for selection		filtering	no. genes showing evidence of natural selection	
	no. total datasets	*p*-value<0.05	no. datasets excl. Med PSSs≥10	*p*-value<0.05	FDR *q*-value<0.10
branch-site model		*ω*_0_≤0, *ω*≥1		*ω*_0_≤0, *ω*>1	*ω*_0_≤0, *ω*>1
ancestral Whippomorpha	6845	106	4974	43	1
hippo terminal branch	6845	201	4974	64	2
ancestral Cetacea	11 925	391	8602	200	6
ancestral Mysticeti	11 925	439	8602	173	15
ancestral Odontoceti	11 925	335	8602	125	11

### 2.4 Network analysis of protein–protein interactions

For genes under positive selection, we built protein–protein interaction networks using Igraph v. 0.7.0 [33]. We interrogated the STRING database [34] with the human and dolphin Ensembl gene identifiers for combined interaction scores. To visualize proteins with common functions, we obtained gene ontology (GO) terms under the biological processes domain using topGO [35]. Corresponding GO term names were exported from Ensembl and AmiGO 2 v. 2.1.4, and GO terms were grouped into functional categories that we predicted to be important during the evolution of cetaceans and hippos (electronic supplementary material, methods). These grouped GO terms were then mapped onto each network.

### 2.5 Test of functional enrichment

For all genes under selection in our focal taxa, regardless of protein–protein interactions, we also tested for functional enrichment using the topGO package [35] (electronic supplementary material, methods, and results and discussion).

## 3. Results and discussion

### 3.1 RNA sequencing, assembly and orthologue identification

The number of candidate one-to-one orthologues across eight whippomorph species ranged from 6249 to 16 047 genes (electronic supplementary material, table S2). We combined annotated CDSs from bottlenose dolphin- and human-anchored blast searches (more than or equal to 50% coverage of the full gene length in the human genome), yielding 9267 and 14 234 one-to-one orthologues, respectively (electronic supplementary material, table S2). By sampling these sequences across additional laurasiatherian mammals, we built 11 925 gene alignments, each of which contained at least one member of each of the two extant cetacean suborders (Mysticeti and Odontoceti). Moreover, over half of these (*n*=6845) also contained the hippo sequence.

### 3.2 Scans for signatures of natural selection

To identify loci under positive selection at a genomic scale, we used branch-site codon models to estimate the ratio of non-synonymous to synonymous substitution rates (d*N*/d*S*) or omega (*ω*) on the ancestral branches of Whippomorpha, Cetacea, Mysticeti and Odontoceti, as well as the terminal hippo branch ((i), (ii), (iii), (iv) and (v), respectively; figure 1). Of 6845 genes containing the hippo sequence, we found 106 genes showing PSSs on the ancestral branch of Whippomorpha, as well as 201 genes on the hippo terminal branch (table 1). Across all 11 925 genes tested, signatures of molecular adaptation were detected in 391 genes on the ancestral branch of Cetacea, compared to 439 and 335 genes on the ancestral branches of Mysticeti and Odontoceti, respectively (table 1). Because signals of positive selection can sometimes arise from alignment errors, for each gene we inspected the distribution of sites with high omega values (*ω*>1) and filtered out genes (*n*=3323) in which such signals were highly aggregated (see Material and methods; table 1; electronic supplementary material, table S3). As a result, the numbers of genes retained as under positive selection decreased to 43 for the ancestral branch of Whippomorpha, and from 201 to 64 for the hippo branch (table 1; electronic supplementary material, table S5). Similarly, excluding genes with highly aggregated PSSs in the three ancestral cetacean branches, we retained 200 genes in the last common ancestor of Cetacea, compared to 173 and 125 genes on the ancestral branches of Mysticeti and Odontoceti, respectively (table 1; electronic supplementary material, table S5). Finally, we applied the FDR; *Q*<0.10) to correct for multiple testing, reducing further the numbers of genes to between one and 15 genes (table 1), although these numbers are likely to be underestimates given our large sample sizes and strict filtering regime.

### 3.3 Functional annotation of genes under selection

**Figure 2.**
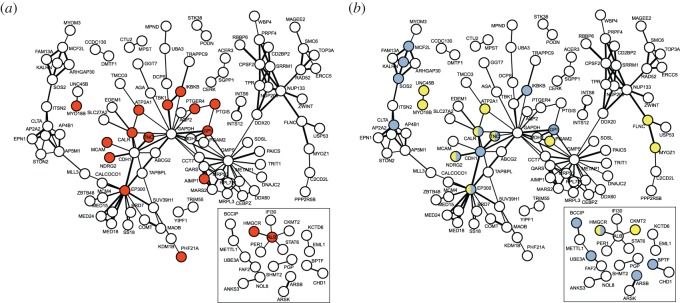
Protein–protein interaction networks for 105 protein-coding gene products tested in both cetaceans and the hippo that were found to be under positive selection in cetaceans. Inset: protein–protein interaction networks for 20 protein-coding genes found to be under positive selection in the hippo. Nodes are labelled with the standard protein names, and the thickness of each connection is scaled to represent the strength of support, with thicker lines representing higher support. (*a*) Highlights proteins involved in the circulatory system (red) and (*b*) highlights proteins involved in muscle (yellow) the nervous system (blue) or both (yellow with blue).

To compare whether sets of genes under selection in the hippo versus cetaceans show broad differences in both functional role and degree of interactions, we plotted interactions among protein products of genes found to be under positive selection (figure 2; electronic supplementary material, methods and figure S1). The hippo network consisted of 20 proteins with at least one interaction, and a key cluster was centred around serum albumin, ALB (see insets figure 2). In comparison, the network constructed for cetaceans comprised more proteins with a greater degree of connectedness; 105 proteins were connected to at least one other protein (figure 2), while several (e.g. GAPDH, EP300, CDH1) had high numbers of connections suggesting that they are important ‘hubs’. The network with the greatest concentration of connections centred around GMPS, a protein involved in guanine synthesis. By mapping GO terms onto these networks, we identified several instances in both taxa in which proteins associated with common tissue types and/or functions were clustered together. For example, in the hippo network, the linked proteins HMGCR and ALB are both associated with circulation (figure 2*a*), whereas in the cetacean network neighbouring proteins with functions related to the nervous system were centred around the hub of CDH1 (figure 2*b*). We also found clustering among proteins involved in other functions; for example, related to cell cycle and ageing in cetaceans (electronic supplementary material, figure S1A), and to lipids in both networks (electronic supplementary material, figure S1B). With one exception, proteins related to hypoxia and DNA repair occurred only in the cetacean network (RAD52, ERCC5 and SMC6), although clustering was limited (electronic supplementary material, figure S1C). No clustering was seen in proteins related to fluid, kidneys, lungs or sensory perception in either network (electronic supplementary material, figure S1D–G). Additional evidence of GO enrichment was restricted to cetaceans, and affected genes involved in brain, blood clotting and sensory perception (electronic supplementary material, results and discussion, tables S7–S9).

### 3.4 Molecular adaptations in the hippo and whippomorph ancestors

Among the 64 genes that were found to have undergone positive selection along the hippo branch (electronic supplementary material, table S5), we found several associated with lipid metabolism, including those with key roles in the biosynthesis and absorption of cholesterol (i.e. *HMGCR*, *CYP2J2* and *CYP8B1*; [36–38]) as well as genes linked to metabolic disorders and/or obesity, including *CPXM1* and *PON3*[39–41]. Other genes seen to be under positive selection in the hippo branch alone are known to function in glucose regulation (e.g. *PDK4*; [42–44]). Interestingly, *PDK4* has also been found to be under selection in another aquatic mammal, the walrus [18]. Another gene with a related metabolic function is *AGL*, involved in glycogen degradation [45,46]. Genes potentially linked to obesity may be related to the hippo's comparatively large size and capacity for fat storage, although the physiology of hippos in general remains little explored [47]. Other genes showing molecular adaptation in the hippo encode proteins that might relate to the unusual demands placed on its circulatory system; notably albumin, a constituent of blood plasma that helps regulate osmotic pressure [48]. Indeed, aside from the need to rapidly cool their skin, hippos appear to experience several circulatory changes during dives, including bradycardia (low heart rate), while at the same time maintaining their arterial blood pressure [49]. We also found positive selection in *PER1*, which encodes an essential component of the circadian clock [50], as well as genes associated with muscle function (e.g. *CKMT2*).

To identify genes important in the early evolution of the Whippomorpha, we examined the ancestral branch and recovered 43 genes under positive selection (electronic supplementary material, tables S5–S6). Of these, we were able to verify PSSs in nine genes for both hippo and cetaceans, as in the rest of the genes hippo sequences contained missing data for amino acid sites identified as being under positive selection. Positively selected genes included *CPT1A*, a gene associated with type II diabetes and involved in fatty acid oxidation, and *XRCC6*, which codes for a DNA repair protein.

### 3.5 Molecular adaptations during cetacean evolution

Like in the hippo, genes underpinning metabolism were also found to be under selection in the cetaceans. Indeed, our results indicate that as many as 25 positively selected genes in cetaceans are involved in sugar metabolism, insulin availability or lipid metabolism. For example, two solute-carrier genes (*SLC5A10* and *SLC9B2*) together with *LMF1* and *MARCH6* have all been implicated in aspects of diabetes, obesity and/or body mass index (electronic supplementary material, table S9). In light of our results, it is noteworthy that the bottlenose dolphin has been proposed as an emerging model for studying type II diabetes based on reports that fasting individuals retain comparatively high glucose levels; this diabetic state may be related to the demand to provide glucose to the brain while diving [3].

Many of the other amino acid changes that we found in the cetacean branches also appear to correspond to their ability to dive and resist oxidative stress. Indeed, some species dive to extraordinary depths; for example, Cuvier's beaked whale (*Ziphius cavirostris*) can reach more than 1000 m [51]. To do this, cetaceans collapse lungs, sequester blood in retia mirabilia and maintain higher haemoglobin and myoglobin concentrations than terrestrial mammals [52]. Our genome-wide scans of nine cetacean species revealed selection in key hypoxia-related genes, including *DDIT4*, *EP300* and *MGEA5*. *MGEA5* interacts directly with the product of *OGT*, a hypoxia gene that has undergone massive gene copy number expansion in cetaceans [23]. We also found evidence for molecular adaptation in at least 13 genes involved in muscle and/or heart development and contraction in cetaceans (electronic supplementary material, table S9). Other studies have reported evidence that cetaceans have developed molecular adaptations to compensate for the lack of oxygen during dives. For example, in many diving aquatic mammals including cetaceans, myoglobin (a protein that stores oxygen in muscles) has evolved to have a greater charge, decreasing the tendency of molecules to clump and therefore increasing oxygen storage capacity in muscle cells [53].

Strikingly, we also found selection in eight genes related to blood clotting or platelet formation. For example, *SERPINC1* produces the protein antithrombin, which interrupts the formation of blood clots [54]. It is notable that *SERPINC1* was identified as being under positive selection in addition to containing a convergent amino acid change in two cetaceans (*T. truncatus* and *O. orca*), the walrus and manatee, in a recent study of marine mammal genomes [18]. Clotting in cetaceans differs from terrestrial mammals in that there is a relative lack of scab formation after wounding [55,56]. This reduced clotting has been attributed to the contact of blood with water rather than air, as well as the need to sequester blood in stagnant reservoirs while diving [56]. Moreover, cetaceans show accelerated wound healing, reducing infection and accelerating tissue repair [57].

We identified signatures of selection in at least 46 genes in the common ancestor of cetaceans associated with the nervous system and brain development, which is interesting given that cetaceans are characterized by large absolute brain sizes, large brain-to-body mass ratios [58], high numbers of neocortical neurons [59] and high cognitive capacity (e.g. [60]). Indeed, many of the genes we found have been implicated in neurological disorders in humans, such as microcephaly, mental retardation, major depressive disorder and Alzheimer's disease (electronic supplementary material). PSSs were also detected in genes involved in myelination [61], neural connectedness, axonal guidance, cognition, neuronal development and neural progenitor cell proliferation. Previous studies have also reported some nervous system genes to be under selection in the *Tursiops* genome [20–22]; however, owing to the number of cetacean taxa included, our study was able to localize positive selection to distinct branches within the tree. In addition, we found that 15 nervous system-related genes showed evidence of positive selection on the mysticete ancestral branch, while six genes contained PSSs on the odontocete ancestral branch. These results are contrary to expectations, as mysticetes, while possessing large absolute brain size in some species, have smaller brain to body size ratios than odontocetes and might be expected to have fewer nervous system genes under selection [58].

The transition from a terrestrial to a wholly aquatic environment means that cetaceans must depend on the properties of water for the transmission of light and sound. Adaptations for living in low light include a thickened cornea, spherical lens and reduced numbers of cones [62]. We determined whether cetaceans show positive selection in loci related to visual perception and found evidence in eight genes, some of which are known to be expressed in the cornea and/or retina, and are otherwise implicated in visual diseases (electronic supplementary material). These results complement earlier findings that cetaceans show several functional molecular changes in (or loss of) their opsin genes [63,64]. We also found molecular adaptation in five genes underpinning hearing; however, despite the fact that the toothed whales have evolved extremely high-frequency sound perception, none of these genes were exclusive to this group. Instead, the gene *TNC* was found to be under selection in the mysticetes only, while *TECTA*, *JAG2* and *USH1C* were under selection in the ancestral branch of all cetaceans (although the latter also showed selection in odontocetes). These hearing genes add to the growing number that has been reported to be of potential importance in whales and dolphins [65–69].

In general, we find a much wider range of molecular adaptations in the cetaceans than hippos, probably reflecting their more derived body plan. For example, apart from those loci already discussed, molecular changes were also found in genes related to the kidneys (*n*=8) and skin/hair (*n*=8) (electronic supplementary material, table S9). Compared to terrestrial mammals, cetacean epidermis typically grows more quickly with fewer layers and is also less keratinized with increased cellular production and lifespan (e.g. [70,71]). Although other studies have identified the integument as a target of molecular evolution in whales and dolphins, these were unable to rule out the possibility that such changes occurred prior to the split with the hippos [20–22,24,72].

When not diving, cetaceans are exposed to the potentially harmful effects of solar radiation [73]. We found many genes under selection that are involved in DNA repair (*n*=5) and/or cancer suppression (*n*=13) in cetaceans, particularly in the mysticete lineage, where we discovered at least 12 of these genes under positive selection, including the *RAD52*, which is essential for double-strand DNA break repair and genomic maintenance of cancer prevention (electronic supplementary material, table S9). Aside from sun exposure, adaptive modification of genes involved in DNA damage repair and tumour suppression may serve to overcome the predicted 1000-fold increase in cancer risk thought to arise as a result of the increased number of cell divisions in these exceptionally large and long-lived mammals [2]. Indeed, Mysticeti contains both the largest (*Balaenoptera musculus*) and oldest recorded (*Balaena mysticetus*; more than 200 years) mammalian species, yet do not show elevated rates of cancer [2]. Our detection of positive selection in DNA damage-related genes in cetaceans augments the result of GO analyses (also see [23,24]).

Overall, we find little support for shared ancestral aquatic adaptations in hippos and cetaceans. In particular, while many molecular adaptations thought to be important for the aquatic environment were recorded on ancestral cetacean branches (mysticetes, odontocetes or both) a comparison of coding sequences that were available for all focal members of the Whippomorpha revealed only a few cases of positive selection along the ancestral branch of the entire clade. Explanations for these findings, apart from the greater degree of morphological adaptation for aquatic existence in cetaceans, include the fragmentary nature of the hippo RNA-sequencing data as well as the relatively short evolutionary time separating the split of Whippomorpha from Ruminantia and the subsequent divergence of hippos from cetaceans. Consequently, our results seem to suggest that cetaceans and hippos evolved most aquatic adaptations separately. On the other hand, we found similar selection pressures acting on genes implicated in lipids in both groups, and more work is needed to determine whether these signatures are related to specialized lipid-rich integuments that characterize semi-aquatic and aquatic animals [13].

## Supplementary Material

Figure S1. Protein-protein interaction networks for 105 protein-coding gene products tested in both cetaceans and the hippo that were found to be under positive selection in the cetaceans. Inset: protein-protein interaction networks for 20 protein-coding genes found to be under positive selection in the hippo. Nodes are labelled with the standard protein names, and the thickness of each connection is scaled to represent the strength of support, with thicker lines representing higher support. Part A highlights proteins involved in the cell cycle and aging (grey); part B highlights proteins involved in lipids (red); Part C in hypoxia and DNA repair (red); parts D-G proteins related to fluid, kidneys, lungs or sensory perception (red) respectively. Supplementary methods. Contains information concerning taxon sampling, sequencing and RNA-Seq de novo assembly, as well as ortholog identification and data set assembly. It also provides additional information for natural selection analyses, GC content estimation, Gene Ontology (GO) enrichment analysis and, finally, network analysis of protein-protein interactions. Supplementary results and discussion. Contains GO analysis results, further grouping of positively selected genes into categories based on organ/organ system or biological process, and comparison of our findings with previous studies. Table S1. RNA extraction QC, RNA-Seq and assembly statistics. Table S2. Ortholog identification. Table S3. Genome-wide analysis for bursts of divergent selection. Table S4. Pearson correlation test between MA model fit (LRT p-values) and ΔGC3 at the third codon position of the branch. Table S7. GO terms enriched for positively selected genes in cetaceans, hippo and/or in both.

## Supplementary Material

Table S5-S6. Separate .xls file with complete lists of genes with PSSs in hippos and whales under branch-site codon and clade models

## Supplementary Material

Table S8. Separate .xls file with results of GO enrichment analysis for the five branches tested for selection for the three GO domains: A. Biological Process; B. Cellular Component; C. Molecular Function.

## Supplementary Material

Table S9. Separate .xls file with a list of a subset of genes under selection grouped by distinct categories, based on organ/organ system or biological process.

## Data accessibility

The new short read data for the hippo and humpback whale have been deposited in the SRA of GenBank under the accession nos. SRR2183469 and SRR2183423. Alignments have been submitted to Dryad: http://dx.doi.org/10.5061/dryad.4cp98.

## References

[1] Gatesy J, Geisler JH, Chang J, Buell C, Berta A, Meredith RW, Springer MS, McGowen MR 2013 A phylogenetic blueprint for a modern whale. Mol. Phyl. Evol. 66, 479–506. (doi:10.1016/j.ympev.2012.10.012) 10.1016/j.ympev.2012.10.012 23103570

[2] Caulin AF, Maley CC 2011 Peto's paradox: evolution's prescription for cancer prevention. Trends Ecol. Evol. 26, 175–182. (doi:10.1016/j.tree.2011.01.002) 21296451 10.1016/j.tree.2011.01.002 PMC3060950

[3] Venn-Watson SK, Ridgway SH 2007 Big brains and blood glucose: common ground for diabetes mellitus in humans and healthy dolphins. Comp. Med. 57, 390–395. 17803054

[4] McGowen MR, Spaulding M, Gatesy J 2009 Divergence date estimation and a comprehensive molecular tree of extant cetaceans. Mol. Phyl. Evol. 53, 891–906. (doi:10.1016/J.Ympev.2009.08.018) 10.1016/j.ympev.2009.08.018 19699809

[5] Meredith RW *et al.* 2011 Impacts of the Cretaceous terrestrial revolution and KPg extinction on mammal diversification. Science 334, 521–524. (doi:10.1126/science.1211028) 21940861 10.1126/science.1211028

[6] Nikaido M, Rooney AP, Okada N 1999 Phylogenetic relationships among cetartiodactyls based on insertions of short and long interspersed elements: hippopotamuses are the closest extant relatives of whales. Proc. Natl Acad. Sci. USA 96, 10 261–10 266. (doi:10.1073/pnas.96.18.10261) 10.1073/pnas.96.18.10261 PMC17876 10468596

[7] Orliac M, Boisserie JR, Maclatchy L, Lihoreau F 2010 Early Miocene hippopotamids (Cetartiodactyla) constrain the phylogenetic and spatiotemporal settings of hippopotamid origin. Proc. Natl Acad. Sci. USA 107, 11 871–11 876. (doi:10.1073/pnas.1001373107) 20547829 10.1073/pnas.1001373107 PMC2900691

[8] Waddell PJ, Okada N, Hasegawa M 1999 Towards resolving the interordinal relationships of placental mammals. Syst. Biol. 48, 1–5. (doi:10.1093/sysbio/48.1.1) 12078634

[9] Montgelard C, Catzeflis FM, Douzery E 1997 Phylogenetic relationships of artiodactyls and cetaceans as deduced from the comparison of cytochrome *b* and 12S rRNA mitochondrial sequences. Mol. Biol. Evol. 14, 550–559. (doi:10.1093/oxfordjournals.molbev.a025792) 9159933 10.1093/oxfordjournals.molbev.a025792

[10] Boisserie JR, Fisher RE, Lihoreau F, Weston EM 2011 Evolving between land and water: key questions on the emergence and history of the Hippopotamidae (Hippopotamoidea, Cetancodonta, Cetartiodactyla). Biol. Rev. 86, 601–625. (doi:10.1111/J.1469-185x.2010.00162.X) 20946539 10.1111/j.1469-185X.2010.00162.x

[11] Coughlin BL, Fish FE 2009 Hippopotamus underwater locomotion: reduced-gravity movements for a massive mammal. J. Mammal. 90, 675–679. (doi:10.1644/08-mamm-a-279r.1)

[12] Luck CP, Wright PG 1964 Aspects of the anatomy and physiology of the skin of the hippopotamus (*H. amphibius*). Exp. Physiol. 49, 1–14. (doi:0.1113/expphysiol.1964.sp001695) 10.1113/expphysiol.1964.sp001695 14115273

[13] Meyer W, Schmidt J, Busche R, Jacob R, Naim HY 2012 Demonstration of free fatty acids in the integument of semi-aquatic and aquatic mammals. Acta Histochem. 114, 145–150. (doi:10.1016/j.acthis.2011.03.011) 21524787 10.1016/j.acthis.2011.03.011

[14] Barklow WE 2004 Amphibious communication with sound in hippos, *Hippopotamus amphibius*. Anim. Behav. 68, 1125–1132. (doi:10.1016/j.anbehav.2003.10.034)

[15] O'Leary MA, Patel BA, Coleman MN 2012 Endocranial petrosal anatomy of Bothriogenys (Mammalia, Artiodactyla, Anthracotheriidae), and petrosal volume and density comparisons among aquatic and terrestrial artiodactyls and outgroups. J. Paleontol. 86, 44–50. (doi:10.1666/10-091.1)

[16] Gatesy J, O'Leary MA 2001 Deciphering whale origins with molecules and fossils. Trends Ecol. Evol. 16, 562–570. (doi:10.1016/S0169-5347(01)02236-4)

[17] Roux J, Privman E, Moretti S, Daub JT, Robinson-Rechavi M, Keller L 2014 Patterns of positive selection in seven ant genomes. Mol. Biol. Evol. 31, 1661–1685. (doi:10.1093/Molbev/Msu141) 24782441 10.1093/molbev/msu141 PMC4069625

[18] Foote AD *et al.* 2015 Convergent evolution of the genomes of marine mammals. Nat. Genet. 47, 272–275. (doi:10.1038/ng.3198) 25621460 10.1038/ng.3198 PMC4644735

[19] Keane M *et al.* 2015 Insights into the evolution of longevity from the bowhead whale genome. Cell Rep. 10, 112–122. (doi:10.1016/j.celrep.2014.12.008) 25565328 10.1016/j.celrep.2014.12.008 PMC4536333

[20] McGowen MR, Grossman LI, Wildman DE 2012 Dolphin genome provides evidence for adaptive evolution of nervous system genes and a molecular rate slowdown. Proc. R. Soc. B 279, 3643–3651. (doi:10.1098/rspb.2012.0869) 10.1098/rspb.2012.0869 PMC3415902 22740643

[21] Nery MF, Gonzalez DJ, Opazo JC 2013 How to make a dolphin: molecular signature of positive selection in cetacean genome. PLoS ONE 8, e65491 (doi:10.1371/journal.pone.0065491) 23840335 10.1371/journal.pone.0065491 PMC3686761

[22] Sun YB, Zhou WP, Liu HQ, Irwin DM, Shen YY, Zhang YP 2013 Genome-wide scans for candidate genes involved in the aquatic adaptation of dolphins. Genome Biol. Evol. 5, 130–139. (doi:10.1093/gbe/evs123) 23246795 10.1093/gbe/evs123 PMC3595024

[23] Yim HS *et al.* 2014 Minke whale genome and aquatic adaptation in cetaceans. Nat. Genet. 46, 88–92. (doi:10.1038/ng.2835) 24270359 10.1038/ng.2835 PMC4079537

[24] Zhou XM *et al.* 2013 Baiji genomes reveal low genetic variability and new insights into secondary aquatic adaptations. Nat. Commun. 4, 2708 (doi:10.1038/ncomms3708) 24169659 10.1038/ncomms3708 PMC3826649

[25] Gui D, Jia KT, Xia J, Yang LL, Chen JL, Wu YP, Yi MS 2013 *De novo* assembly of the Indo-Pacific humpback dolphin leucocyte transcriptome to identify putative genes involved in the aquatic adaptation and immune response. PLoS ONE 8, e72417 (doi:10.1371/journal.pone.0072417) 24015242 10.1371/journal.pone.0072417 PMC3756080

[26] Lindblad-Toh K *et al.* 2011 A high-resolution map of human evolutionary constraint using 29 mammals. Nature 478, 476–482. (doi:10.1038/nature10530) 21993624 10.1038/nature10530 PMC3207357

[27] Kinsella RJ *et al.* 2011 Ensembl BioMarts: a hub for data retrieval across taxonomic space. Database 2011, bar030. (doi:10.1093/database/bar030) 10.1093/database/bar030 PMC3170168 21785142

[28] Loytynoja A, Goldman N 2005 An algorithm for progressive multiple alignment of sequences with insertions. Proc. Natl Acad. Sci. USA 102, 10 557–10 562. (doi:10.1073/pnas.0409137102) 10.1073/pnas.0409137102 PMC1180752 16000407

[29] Penn O, Privman E, Ashkenazy H, Landan G, Graur D, Pupko T 2010 GUIDANCE: a web server for assessing alignment confidence scores. Nuclic Acid Res. 38, W23–W28. (doi:10.1093/nar/gkq443) 10.1093/nar/gkq443 PMC2896199 20497997

[30] Yang ZH 2007 PAML 4: phylogenetic analysis by maximum likelihood. Mol. Biol. Evol. 24, 1586–1591. (doi:10.1093/Molbev/Msm088) 17483113 10.1093/molbev/msm088

[31] Zhang JZ, Nielsen R, Yang ZH 2005 Evaluation of an improved branch-site likelihood method for detecting positive selection at the molecular level. Mol. Biol. Evol. 22, 2472–2479. (doi:10.1093/Molbev/Msi237) 16107592 10.1093/molbev/msi237

[32] Benjamini Y, Hochberg Y 1995 Controlling the false discovery rate: a practical and powerful approach to multiple testing. J. R. Stat. Soc. B 57, 289–300.

[33] Csardi G, Nepusz T 2006 The igraph software package for complex network research. InterJournal Complex Systems, 1695 See http://igraph.org

[34] Szklarczyk D *et al.* 2015 STRING v10: protein–protein interaction networks, integrated over the tree of life. Nuclic Acid Res. 43, D447–D452. (doi:10.1093/nar/gku1003) 10.1093/nar/gku1003 PMC4383874 25352553

[35] Alexa A, Rahnenfuhrer J, Lengauer T 2006 Improved scoring of functional groups from gene expression data by decorrelating GO graph structure. Bioinformatics 22, 1600–1607. (doi:10.1093/bioinformatics/btl140) 16606683 10.1093/bioinformatics/btl140

[36] Lindgren V, Luskey KL, Russell DW, Francke U 1985 Human genes involved in cholesterol- metabolism: chromosomal mapping of the loci for the low-density-lipoprotein receptor and 3-hydroxy-3-methylglutaryl-coenzyme-A reductase with CDNA probes. Proc. Natl Acad. Sci. USA 82, 8567–8571. (doi:10.1073/pnas.82.24.8567) 3866240 10.1073/pnas.82.24.8567 PMC390958

[37] Yang YZ, Eggertsen G, Gafvels M, Andersson U, Einarsson C, Bjorkhem I, Chiang JYL 2004 Mechanisms of cholesterol and sterol regulatory element binding protein regulation of the sterol 12 *α*-hydroxylase gene (*CYP8B1*). Biochem. Biophys. Res. Commun. 320, 1204–1210. (doi:10.1016/j.bbrc.2004.06.069) 15249218 10.1016/j.bbrc.2004.06.069

[38] Wu SN, Zhang Y, Gardner CO, Chen Q, Li Y, Wang GL, Gao PJ, Zhu DL 2007 Evidence for association of polymorphisms in *CYP2J2* and susceptibility to essential hypertension. Ann. Hum. Genet. 71, 519–525. (doi:10.1111/J.1469-1809.2007.00346.X) 17286575 10.1111/j.1469-1809.2007.00346.x

[39] Maier EM *et al.* 2005 Population spectrum of *ACADM* genotypes correlated to biochemical phenotypes in newborn screening for medium-chain acyl-CoA dehydrogenase deficiency. Hum. Mutat. 25, 443–452. (doi:10.1002/humu.20163) 15832312 10.1002/humu.20163

[40] Perez-Montarelo D, Madsen O, Alves E, Rodriguez MC, Folch JM, Noguera JL, Groenen MAM, Fernandez AI 2014 Identification of genes regulating growth and fatness traits in pig through hypothalamic transcriptome analysis. Phys. Genomics 46, 195–206. (doi:10.1152/physiolgenomics.00151.2013) 10.1152/physiolgenomics.00151.2013 PMC3949103 24280257

[41] Rai MF, Patra D, Sandell LJ, Brophy RH 2014 Relationship of gene expression in the injured human meniscus to body mass index a biologic connection between obesity and osteoarthritis. Arthritis Rheumatol. 66, 2152–2164. (doi:10.1002/art.38643) 24692131 10.1002/art.38643 PMC4116431

[42] Wende AR, Huss JM, Schaeffer PJ, Giguere V, Kelly DP 2005 PGC-1 *α* coactivates PDK4 gene expression via the orphan nuclear receptor ERR *α*: a mechanism for transcriptional control of muscle glucose metabolism. Mol. Cell Biol. 25, 10 684–10 694. (doi:10.1128/mcb.25.24.10684-10694.2005) 10.1128/MCB.25.24.10684-10694.2005 PMC1316952 16314495

[43] Connaughton S, Chowdhury F, Attia RR, Song SL, Zhang Y, Elam MB, Cook GA, Park EA 2010 Regulation of pyruvate dehydrogenase kinase isoform 4 (*PDK4*) gene expression by glucocorticoids and insulin. Mol. Cell Endocrinol. 315, 159–167. (doi:10.1016/j.mce.2009.08.011) 19703515 10.1016/j.mce.2009.08.011 PMC2815206

[44] Rodbell M, Birnbaum L, Pohl SL, Sundby F 1971 Reaction of glucagon with its receptor: evidence for discrete regions of activity and binding in glucagon molecule. Proc. Natl Acad. Sci. USA 68, 909–913. (doi:10.1073/pnas.68.5.909) 5280527 10.1073/pnas.68.5.909 PMC389078

[45] Horinishi A, Okubo M, Tang NLS, Hui J, To KF, Mabuchi T, Okada T, Mabuchi H, Murase T 2002 Mutational and haplotype analysis of AGL in patients with glycogen storage disease type III. J. Hum. Genet. 47, 55–59. (doi:10.1007/S100380200000) 11924557 10.1007/s100380200000

[46] Fujita Y *et al.* 2004 Hypercholesterolemia associated with splice-junction variation of inter-*α*-trypsin inhibitor heavy chain 4 (*ITIH4*) gene. J. Hum. Genet. 49, 24–28. (doi:10.1007/s10038-003-0101-8) 14661079 10.1007/s10038-003-0101-8

[47] Eltringham SK 1999 The hippos: natural history and conservation. Princeton, NJ: Princeton University Press.

[48] Ajioka RS, Phillips JD, Kushner JP 2006 Biosynthesis of heme in mammals. Biochim.Biophys. Acta Mol. Cell. Res. 1763, 723–736. (doi:10.1016/J.Bbamcr.2006.05.005) 10.1016/j.bbamcr.2006.05.005 16839620

[49] Elsner R, Franklin DL, Van Citters RL, Kenney DW 1966 Cardiovascular defense against asphyxia. Science 153, 941–949. (doi:10.1126/science.153.3739.941) 5917556 10.1126/science.153.3739.941

[50] Reppert SM, Weaver DR 2002 Coordination of circadian timing in mammals. Nature 418, 935–941. (doi:10.1038/nature00965) 12198538 10.1038/nature00965

[51] Tyack PL, Johnson M, Soto NA, Sturlese A, Madsen PT 2006 Extreme diving of beaked whales. J. Exp. Biol. 209, 4238–4253. (doi:10.1242/Jeb.02505) 17050839 10.1242/jeb.02505

[52] Kooyman GL, Ponganis PJ 1998 The physiological basis of diving to depth: birds and mammals. Annu. Rev. Physiol. 60, 19–32. (doi:10.1146/Annurev.Physiol.60.1.19) 9558452 10.1146/annurev.physiol.60.1.19

[53] Mirceta S, Signore AV, Burns JM, Cossins AR, Campbell KL, Berenbrink M 2013 Evolution of mammalian diving capacity traced by myoglobin net surface charge. Science 340, 1234192 (doi:10.1126/science.1234192) 23766330 10.1126/science.1234192

[54] Picard A, Oger PM, Daniel I, Cardon H, Montagnac G, Chervin JC 2006 A sensitive pressure sensor for diamond anvil cell experiments up to 2 GPa: FluoSpheres^r^. J. Appl. Phys. 100, 034915, (doi:10.1063/1.2234821)

[55] Bruceallen LJ, Geraci JR 1985 Wound-healing in the bottlenose dolphin (*Tursiops truncatus*). Can. J. Fish Aquat. Sci. 42, 216–228. (doi:10.1139/F85-029)

[56] Tibbs RF, Elghetany MT, Tran LT, Van Bonn W, Romano T, Cowan DF 2005 Characterization of the coagulation system in healthy dolphins: the coagulation factors, natural anticoagulants, and fibrinolytic products. Comp. Clin. Pathol. 14, 95–98. (doi:10.1007/s00580-005-0567-1)

[57] Griffeth RJ, Garcia-Parraga D, Mellado-Lopez M, Crespo-Picazo JL, Soriano-Navarro M, Martinez-Romero A, Moreno-Manzano V 2014 Platelet-rich plasma and adipose-derived mesenchymal stem cells for regenerative medicine-associated treatments in bottlenose dolphins (*Tursiops truncatus*). PLoS ONE 9, e108439 (doi:10.1371/journal.pone.0108439) 25251412 10.1371/journal.pone.0108439 PMC4177220

[58] Montgomery SH, Geisler JH, McGowen MR, Fox C, Marino L, Gatesy J 2013 The evolutionary history of cetacean brain and body size. Evolution 67, 3339–3353. (doi:10.1111/evo.12197) 24152011 10.1111/evo.12197

[59] Mortensen HS, Pakkenberg B, Dam M, Dietz R, Sonne C, Mikkelsen B, Eriksen N 2014 Quantitative relationships in delphinid neocortex. Front. Neuroanat. 8, 132 (doi:10.3389/fnana.2014.00132) 25505387 10.3389/fnana.2014.00132 PMC4244864

[60] Marino L *et al.* 2007 Cetaceans have complex brains for complex cognition. PLoS Biol. 5, e139 (doi:10.1371/journal.pbio.0050139) 17503965 10.1371/journal.pbio.0050139 PMC1868071

[61] Noseda R *et al.* 2013 Ddit4/Redd1/Rtp801 is a novel negative regulator of Schwann cell myelination.J. Neurosci. 33, 15 295–15 305. (doi:10.1523/JNEUROSCI.2408-13.2013) 10.1523/JNEUROSCI.2408-13.2013 PMC3988321 24048858

[62] Mass AM, Supin AY 2007 Adaptive features of aquatic mammals' eye. Anat. Rec. 290, 701–715. (doi:10.1002/ar.20529) 10.1002/ar.20529 17516421

[63] Meredith RW, Gatesy J, Emerling CA, York VM, Springer MS 2013 Rod monochromacy and the coevolution of cetacean retinal opsins. PLoS Genet. 9, e1003432 (doi:10.1371/journal.pgen.1003432) 23637615 10.1371/journal.pgen.1003432 PMC3630094

[64] Zhao H, Ru B, Teeling EC, Faulkes CG, Zhang S, Rossiter SJ 2009 Rhodopsin molecular evolution in mammals inhabiting low light environments. PLoS ONE 4, e8326 (doi:10.1371/journal.pone.0008326) 20016835 10.1371/journal.pone.0008326 PMC2790605

[65] Davies KT, Cotton JA, Kirwan JD, Teeling EC, Rossiter SJ 2012 Parallel signatures of sequence evolution among hearing genes in echolocating mammals: an emerging model of genetic convergence. Heredity 108, 480–489. (doi:10.1038/hdy.2011.119) 22167055 10.1038/hdy.2011.119 PMC3330687

[66] Li Y, Liu Z, Shi P, Zhang J 2010 The hearing gene *Prestin* unites echolocating bats and whales. Curr. Biol. 20, R55–R56. (doi:10.1016/j.cub.2009.11.042) 20129037 10.1016/j.cub.2009.11.042 PMC11646320

[67] Liu Y, Rossiter SJ, Han X, Cotton JA, Zhang S 2010 Cetaceans on a molecular fast track to ultrasonic hearing. Curr. Biol. 20, 1834–1839. (doi:10.1016/j.cub.2010.09.008) 20933423 10.1016/j.cub.2010.09.008

[68] Parker J, Tsagkogeorga G, Cotton JA, Liu Y, Provero P, Stupka E, Rossiter SJ 2013 Genome-wide signatures of convergent evolution in echolocating mammals. Nature 502, 228–231. (doi:10.1038/nature12511) 24005325 10.1038/nature12511 PMC3836225

[69] Shen YY, Liang L, Li GS, Murphy RW, Zhang YP 2012 Parallel evolution of auditory genes for echolocation in bats and toothed whales. PLoS Genet. 8, e1002788 (doi:10.1371/journal.pgen.1002788) 22761589 10.1371/journal.pgen.1002788 PMC3386236

[70] Hicks BD, St Aubin DJ, Geraci JR, Brown WR 1985 Epidermal growth in the bottlenose dolphin, *Tursiops truncatus*. J. Invest. Dermatol. 85, 60–63. (doi:10.1111/1523-1747.ep12275348) 4008976 10.1111/1523-1747.ep12275348

[71] Reeb D, Best PB, Kidson SH 2007 Structure of the integument of southern right whales, *Eubalaena australis*. Anat. Rec. 290, 596–613. (doi:10.1002/ar.20535) 10.1002/ar.20535 17516424

[72] Chen Z, Wang Z, Xu S, Zhou K, Yang G 2013 Characterization of hairless (*Hr*) and *FGF5* genes provides insights into the molecular basis of hair loss in cetaceans. BMC Evol. Biol. 13, 34 (doi:10.1186/1471-2148-13-34) 23394579 10.1186/1471-2148-13-34 PMC3608953

[73] Martinez-Levasseur LM, Gendron D, Knell RJ, O'Toole EA, Singh M, Acevedo-Whitehouse K 2011 Acute sun damage and photoprotective responses in whales. Proc R. Soc. B. 278, 1581–1586. (doi:10.1098/rspb.2010.1903) 10.1098/rspb.2010.1903 PMC3081749 21068035

