# Supplementary material for: A phylogenomic analysis of the role and timing of molecular adaptation in the aquatic transition of cetartiodactyl mammals
Source: R Soc Open Sci. 2015 Sep 30;2(9):150156. doi: 10.1098/rsos.150156 (PMC4593674; doi:10.1098/rsos.150156)
Supplement: Figure S1. Protein-protein interaction networks for 105 protein-coding gene products tested in both cetaceans and the hippo that were found to be under positive selection in the cetaceans. Inset: protein-protein interaction networks for 20 protein-coding genes found to be under positive selection in [file rsos150156supp1.pdf]

DATA SUPPLEMENT

A phylogenomic analysis of the role and timing of molecular adaptation in the aquatic transition of cetartiodactyl mammals.

Georgia Tsagkogeorga, Michael R. McGowen, Kalina T.J. Davies, Simon Jarman, Andrea Polanowski, Mads F. Bertelsen and Stephen J. Rossiter

CONTENTS:

Figure S1. Protein-protein interaction networks for 107 protein-coding gene products tested in both cetaceans and the hippo that were found to be under positive selection in the cetaceans. Inset: protein-protein interaction networks for 20 protein-coding genes found to be under positive selection in the hippo. Nodes are labelled with the standard protein names, and the thickness of each connection is scaled to represent the strength of support, with thicker lines representing higher support. Part A highlights proteins involved in the cell cycle and aging (grey); part B highlights proteins involved in lipids (red); Part C in hypoxia and DNA repair (red); parts D-F proteins related to fluid, kidneys, lungs or sensory perception (red) respectively.

Supplementary material. Contains information concerning taxon sampling, sequencing and RNA-Seq *de novo* assembly, as well as ortholog identification and data set assembly. It also provides additional information for natural selection analyses, GC content estimation, Gene Ontology (GO) enrichment analysis and, finally, network analysis of protein-protein interactions.

Table S1. RNA extraction QC, RNA-Seq and assembly statistics.
Table S2. Ortholog identification.
Table S3. Genome-wide analysis for bursts of divergent selection
Table S4. Pearson correlation test between MA model fit (LRT *p-values*) and ΔGC3 at the third codon position of the branch.
Table S5-S6. Separate .xls file with complete lists of genes with PSSs in hippos and whales under branch-site codon and clade models.
Table S7. GO terms enriched for positively selected genes in cetaceans, hippo and/or in both.
Table S8. Separate .xls file with results of GO enrichement analysis for the five branches tested for selection for the three GO domains: A. Biological Process; B. Cellular Component; C. Molecular Function.

Figure S1

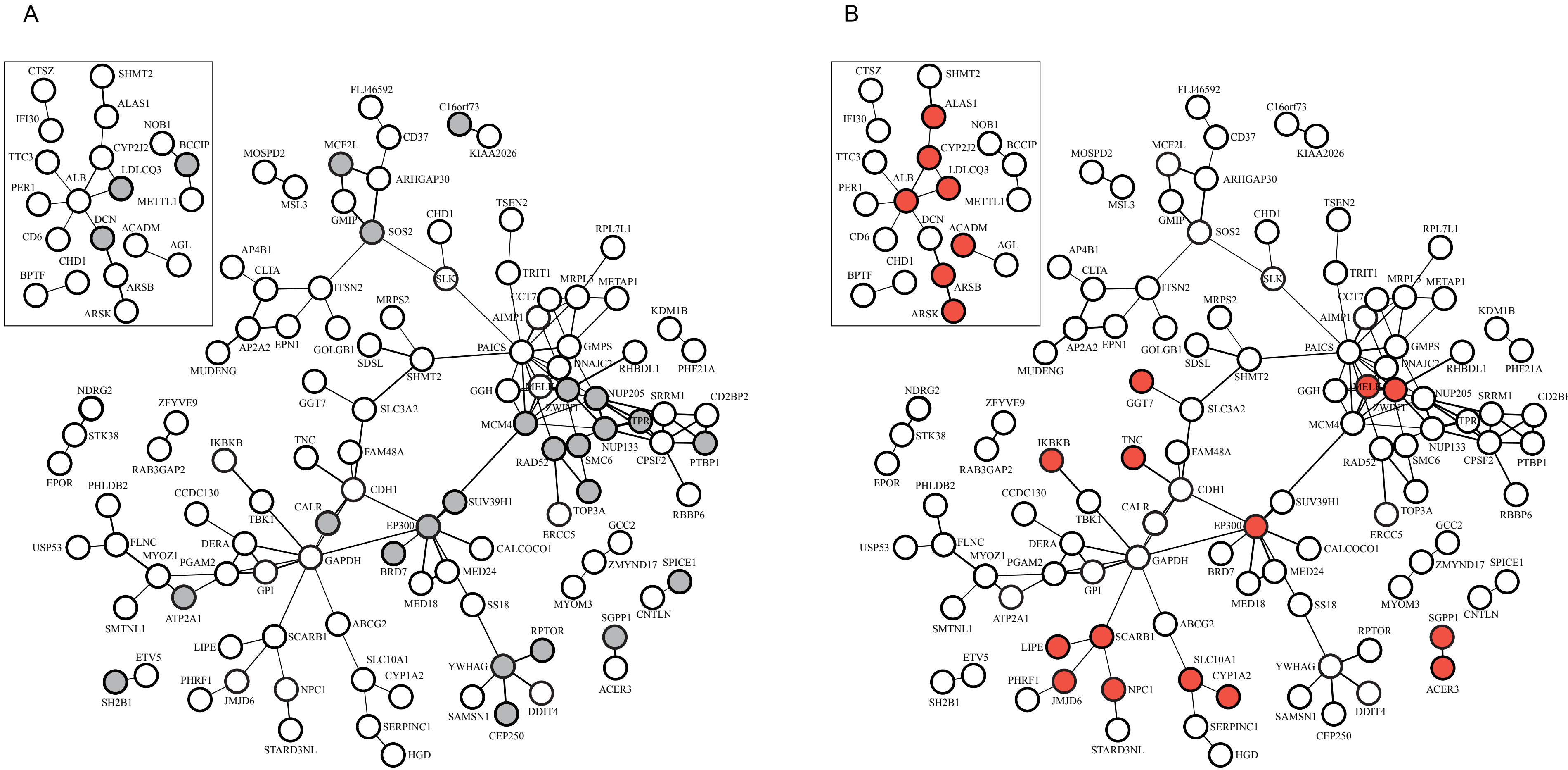
A
B
CTSZ
SHMT2
ALAS1
IFI30
NOB1
BCCIP
TTC3
CYP2J2
LDLCQ3
ALB
PER1
METTL1
DCN
ACADM
CD6
AGL
CHD1
BPTF
ARSB
ARSK
FLJ46592
C16orf73
CD37
KIAA2026
MOSPD2
MCF2L
ARHGAP30
MSL3
GMIP
SOS2
CHD1
TSEN2
AP4B1
CLTA
ITSN2
SLK
TRIT1
RPL7L1
CCT7
MRPL3
METAP1
MRPS2
AIMP1
AP2A2
EPN1
GOLGB1
KDM1B
MUDENG
SDSL
PAICS
GMPS
PHF21A
RHBDL1
SHMT2
DNAJC2
NDRG2
GGT7
GGH
MELK
NUP205
SRRM1
CD2BP2
ZFYVE9
SLC3A2
ZWINT
STK38
MCM4
TPR
NUP133
CPSF2
PTBP1
IKBKB
TNC
FAM48A
RAB3GAP2
RAD52
SMC6
EPOR
PHLDB2
CDH1
CCDC130
TBK1
SUV39H1
TOP3A
RBBP6
CALR
ERCC5
USP53
FLNC
DERA
EP300
GCC2
MYOZ1
PGAM2
GAPDH
CALCOCO1
ZMYND17
SPICE1
BRD7
GPI
MED24
ATP2A1
MED18
MYOM3
CNTLN
SMTNL1
SS18
ABCG2
SGPP1
RPTOR
LIPE
SCARB1
ETV5
SLC10A1
YWHAG
PHRF1
CYP1A2
JMJD6
NPC1
ACER3
SH2B1
SAMSN1
DDIT4
SERPINC1
STARD3NL
CEP250
HGD

C

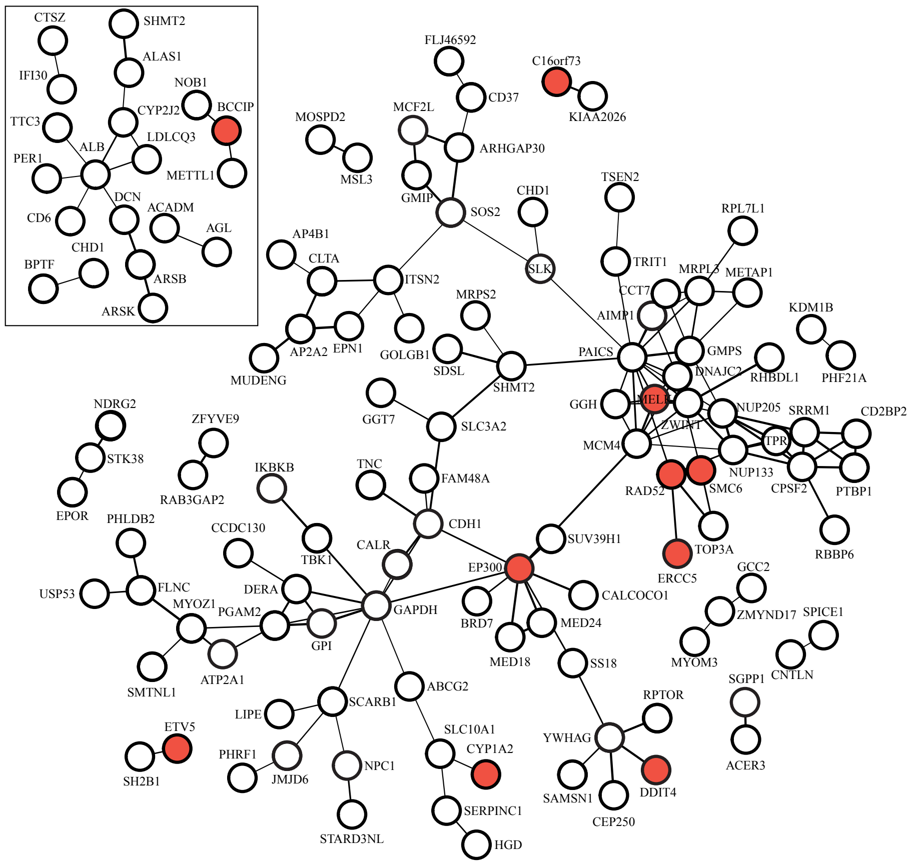
CTSZ
SHMT2
IFI30
ALAS1
NOB1
BCCIP
TTC3
CYP2J2
ALB
LDLCQ3
PER1
METTL1
CD6
DCN
ACADM
AGL
BPTF
CHD1
ARSB
ARSK
FLJ46592
C16orf73
CD37
KIAA2026
MOSPD2
MCF2L
ARHGAP30
MSL3
GMIP
SOS2
CHD1
TSEN2
AP4B1
CLTA
ITSN2
SLK
RPL7L1
TRIT1
MRPL3
METAP1
CCT7
MRPS2
AIMP1
KDM1B
AP2A2
EPN1
GOLGB1
SDSL
PAICS
GMPS
MUDENG
SHMT2
DNAJC2
RHBDL1
PHF21A
NDRG2
GGH
MELK
ZFYVE9
GGT7
SLC3A2
NUP205
SRRM1
CD2BP2
ZWINT
STK38
MCM4
TPR
NUP133
IKBKB
TNC
FAM48A
RAD52
SMC6
CPSF2
PTBP1
RAB3GAP2
EPOR
PHLDB2
CCDC130
CDH1
TBK1
SUV39H1
TOP3A
RBBP6
CALR
EP300
ERCC5
GCC2
USP53
FLNC
DERA
CALCOCO1
MYOZ1
PGAM2
GAPDH
ZMYND17
SPICE1
BRD7
MED24
GPI
MYOM3
CNTLN
ATP2A1
MED18
SS18
SMTNL1
SGPP1
ABCG2
RPTOR
ETV5
LIPE
SCARB1
SLC10A1
YWHAG
PHRF1
CYP1A2
NPC1
JMJD6
ACER3
SH2B1
SAMSN1
DDIT4
SERPINC1
CEP250
STARD3NL
HGD

D

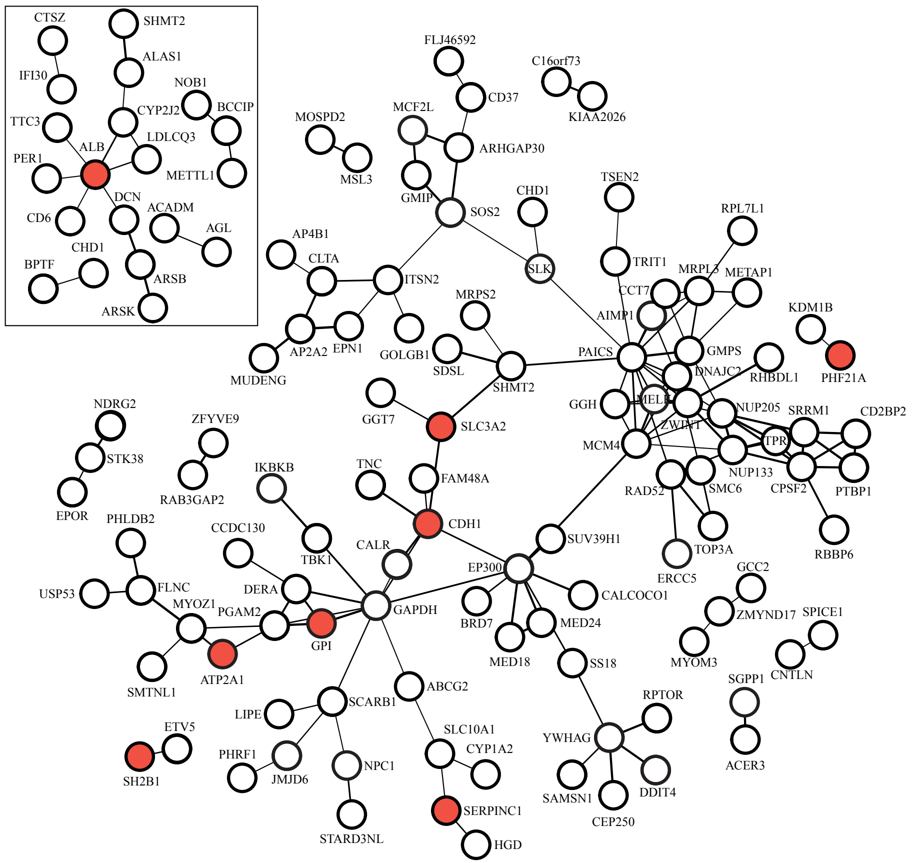
CTSZ
SHMT2
IFI30
ALAS1
NOB1
BCCIP
TTC3
CYP2J2
ALB
LDLCQ3
PER1
METTL1
CD6
DCN
ACADM
AGL
BPTF
CHD1
ARSB
ARSK
FLJ46592
C16orf73
CD37
KIAA2026
MOSPD2
MCF2L
ARHGAP30
MSL3
GMIP
SOS2
CHD1
TSEN2
AP4B1
CLTA
ITSN2
SLK
RPL7L1
TRIT1
MRPL3
METAP1
CCT7
MRPS2
AIMP1
KDM1B
AP2A2
EPN1
GOLGB1
SDSL
PAICS
GMPS
MUDENG
SHMT2
DNAJC2
RHBDL1
PHF21A
NDRG2
GGH
MELK
ZFYVE9
GGT7
SLC3A2
NUP205
SRRM1
CD2BP2
ZWINT
STK38
MCM4
TPR
NUP133
IKBKB
TNC
FAM48A
RAD52
SMC6
CPSF2
PTBP1
RAB3GAP2
EPOR
PHLDB2
CCDC130
CDH1
TBK1
SUV39H1
TOP3A
RBBP6
CALR
EP300
ERCC5
GCC2
USP53
FLNC
DERA
CALCOCO1
MYOZ1
PGAM2
GAPDH
ZMYND17
SPICE1
BRD7
MED24
GPI
MYOM3
CNTLN
ATP2A1
MED18
SS18
SMTNL1
SGPP1
ABCG2
RPTOR
ETV5
LIPE
SCARB1
SLC10A1
YWHAG
PHRF1
CYP1A2
NPC1
JMJD6
ACER3
SH2B1
SAMSN1
DDIT4
SERPINC1
CEP250
STARD3NL
HGD

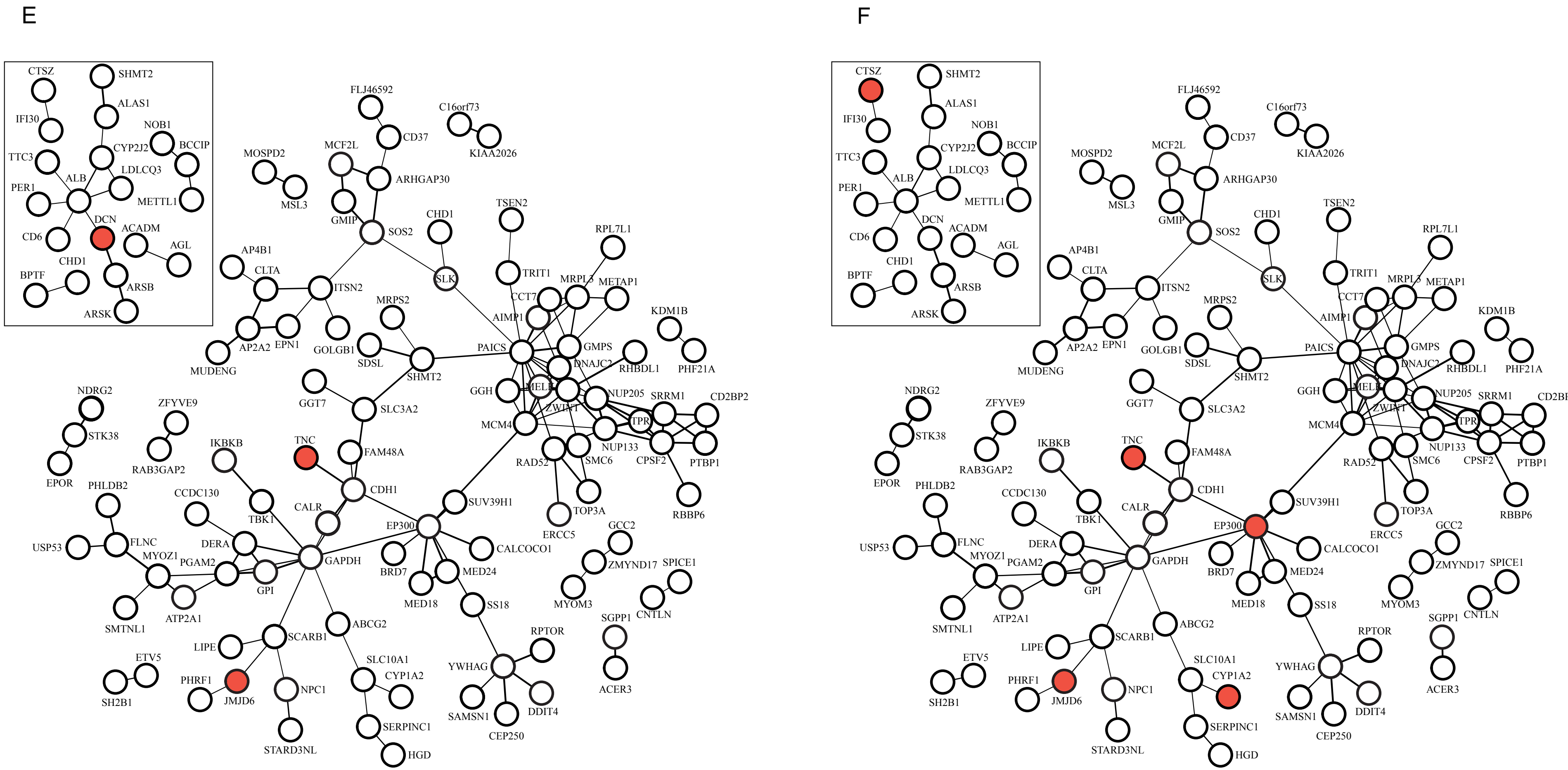
E
F
CTSZ
IFI30
SHMT2
ALAS1
NOB1
BCCIP
TTC3
CYP2J2
LDLCQ3
ALB
PER1
METTL1
DCN
ACADM
CD6
AGL
BPTF
CHD1
ARSB
ARSK
FLJ46592
C16orf73
CD37
KIAA2026
MOSPD2
MCF2L
ARHGAP30
MSL3
GMIP
SOS2
CHD1
TSEN2
AP4B1
SLK
RPL7L1
CLTA
ITSN2
TRIT1
MRPL3
METAP1
CCT7
MRPS2
AIMP1
KDM1B
AP2A2
EPN1
GOLGB1
PAICS
GMPS
SDSL
DNAJC2
PHF21A
MUDENG
SHMT2
RHBDL1
NDRG2
GGT7
GGH
MELK
NUP205
SRRM1
CD2BP2
ZFYVE9
SLC3A2
ZWINT
STK38
MCM4
TPR
NUP133
TNC
IKBKB
FAM48A
RAD52
SMC6
CPSF2
PTBP1
RAB3GAP2
EPOR
PHLDB2
CDH1
CCDC130
SUV39H1
TOP3A
RBBP6
TBK1
CALR
EP300
ERCC5
GCC2
USP53
FLNC
DERA
CALCOCO1
MYOZ1
PGAM2
GAPDH
BRD7
ZMYND17
SPICE1
GPI
MED24
SMTNL1
ATP2A1
MED18
SS18
MYOM3
CNTLN
SGPP1
ABCG2
RPTOR
LIPE
SCARB1
ETV5
SLC10A1
CYP1A2
YWHAG
ACER3
PHRF1
NPC1
SH2B1
JMJD6
SERPINC1
SAMSN1
DDIT4
CEP250
STARD3NL
HGD

G

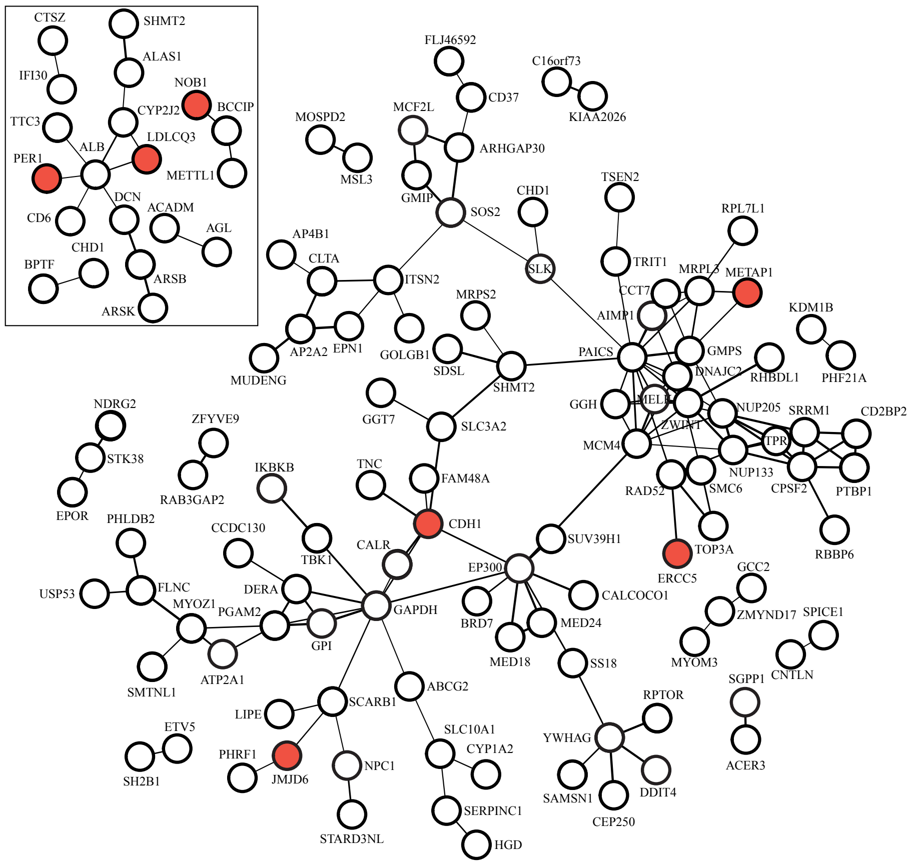
CTSZ
SHMT2
ALAS1
IFI30
NOB1
CYP2J2
BCCIP
TTC3
LDLCQ3
ALB
PER1
METTL1
DCN
ACADM
CD6
AGL
CHD1
BPTF
ARSB
ARSK
FLJ46592
C16orf73
CD37
KIAA2026
MOSPD2
MCF2L
ARHGAP30
MSL3
GMIP
SOS2
CHD1
TSEN2
RPL7L1
AP4B1
CLTA
ITSN2
SLK
TRIT1
MRPS2
CCT7
MRPL3
METAP1
AIMP1
KDM1B
AP2A2
EPN1
GOLGB1
GMPS
PHF21A
MUDENG
SDSL
PAICS
DNAJC2
RHBDL1
SHMT2
GGH
ZWINT
NUP205
SRRM1
CD2BP2
GGT7
SLC3A2
MCM4
TPR
NUP133
NDRG2
ZFYVE9
STK38
IKBKB
TNC
FAM48A
RAD52
SMC6
CPSF2
PTBP1
RAB3GAP2
EPOR
PHLDB2
CCDC130
CDH1
TBK1
CALR
SUV39H1
TOP3A
RBBP6
ERCC5
EP300
GCC2
USP53
FLNC
DERA
MYOZ1
PGAM2
GAPDH
BRD7
CALCOCO1
ZMYND17
SPICE1
GPI
MED24
SMTNL1
ATP2A1
MED18
MYOM3
CNTLN
SS18
ABCG2
SGPP1
SCARB1
RPTOR
LIPE
ETV5
SLC10A1
CYP1A2
YWHAG
PHRF1
NPC1
SH2B1
JMJD6
SAMSN1
DDIT4
ACER3
SERPINC1
CEP250
STARD3NL
HGD

Supplementary material

Taxon sampling, sequencing and RNA-Seq *de novo* assembly. We obtained new RNA-Seq data from the common hippo *H. amphibius* as well as from the humpback whale *M. novaeangliae*. For the hippo, we used a pool of tissue types (muscle, skin, heart, liver, spleen, kidney, lung and neural tissue) obtained from an adult female euthanized at Copenhagen Zoo. For the humpback whale, we pooled skin biopsies from three adult males. RNA was isolated at BGI (hippo) and the Australian Marine Mammal Centre (whale). cDNA library construction followed by pair-end Illumina HiSeq sequencing was performed at BGI (see Additional file 2). CDS data from three additional cetacean species, the minke whale *B. acutorostrata*, the fin whale *B. physalus*, and the finless porpoise *Neophocaena phocaenoides* were from Yim et al (2014). RNA-Seq sequences for the sperm whale *Physeter macrocephalus* and the Indo-Pacific humpback dolphin *Sousa chinensis*, were downloaded from the SRA (Short Read Archive) of GenBank (Acc. Nos. SRX220350-SRX220358 and ERX283216, respectively). Similar to the Illumina data from the humpback whale and hippo, all RNA-Seq data were assembled into transcripts *de novo* using the program Trinity (trinityrnaseq-r2013-02-25) under the default parameters [1]. For the bottlenose dolphin *T. truncatus* and the killer whale *Orcinus orca*, assembled and annotated RNA transcripts were previously available and downloaded directly from the Ensembl database (release 70) and Genbank, respectively. In the case of the river dolphin *Lipotes vexillifer*, there were no transcriptome data available at the time, so we used the software MAKER2 [2] to perform a *de novo* gene annotation on its genome available in Genbank.

Ortholog identification and data set assembly. To build clusters of orthologous CDSs across all sampled cetaceans and the hippo, we performed a series of reciprocal similarity searches using blastx and tblastn. We used the bottlenose dolphin and human genomes as a reference and blasted the longest protein product of each locus (~16,500 sequences in the dolphin and ~23,600 in the human genomes) against our assembled sequences for each species. Hits in the targeted sequence pool were next blasted back to the initial *T. truncatus* and *H. sapiens* proteins, and only reciprocal matches were retained as putatively orthologous sequences. Based on orthologs that we successfully identified in at least one Mysticeti and one Odontoceti species, we next populated our gene sets with single copy orthologous (one-to-one) coding sequences of other (lauriasiatherian) mammals using a custom perl script in Ensembl API [3]. The number of candidate one-to-one orthologs across eight cetancodontan species ranged from 6,249 to 16,047 genes (see Additional file 2). More orthologs were obtained in human-anchored similarity searches, despite the fact that the bottlenose dolphin is a cetacean; this reflects the better quality of the human genome. One exception was the finless porpoise, due to the CDS data for this species being generated indirectly by SNP calling against bottlenose dolphin genomic sequence [4]. Overall, transcriptome sequencing yielded fewer complete CDS sequences compared to genome-based annotation methods (gene prediction or SNP calling). For example, killer whale CDSs derived from genomic data were identified for 7,981 human orthologs, whereas humpback whale CDSs derived from the transcriptome sequenced here matched 2,396 human CDSs.

The collected CDSs of each locus were next aligned as codons using PRANK v.130820 [5] and multiple sequence alignment (MSA) reliability was assessed using GUIDANCE [6]. GUIDANCE assigns scores (from 0 to 1) by comparing a set of MSAs generated by the original alignment algorithm based on bootstrap guide-trees, here 10. When a CDS alignment contained sequences with a score lower than 0.6,

these were pruned from the dataset as unreliable and a new MSA was re-built from the reduced data under the original parameters, as above. Codon sites below a score of 0.93 (GUIDANCE default value) were discarded, as well as sites with gaps in more than 50% of the sequences sampled in the data set. We observed that, in many alignments, the CDS of the minke whale presented long internal deletions, possibly the result of missing exons excluded from the original annotations using gene prediction analyses [4], the accuracy of which for identifying genes in draft genomes is known to be prone to errors [7]. To account for this type of error in our data, regions with deletions in the *B. acutorostrata* sequences were removed from the alignment, plus 10 flanking codon positions upstream and downstream of the indel. Finally, all sequences were trimmed by 10 codons at both the 5' and 3' ends.

Natural selection analyses. In addition to the branch-site model MA, we also implemented the clade model C [8, 9] to test for divergent selection pressures acting on one of the following groups in the Laurasiatheria tree [10, 11]: (I) Cetancodonta (Hippopotamidae + Cetacea); (II) Cetacea; (III) Mysticeti and (IV) Odontoceti (Figure 1). Clade model C assumes three classes of sites, which differ in their selection pressure, as measured by dN/dS or ω. In the first two site classes, ω was constrained to be under purifying selection ($0 < \omega_0 < 1$) or neutral ($\omega_1 = 1$), while in the third class, ω was estimated separately in foreground (ω_2) and background (ω_3) branches without constraint. Likelihood ratio tests (LRT) were used to assess the model fit of the clade models over the null model M1a (Nearly Neutral), in which two sites are constrained to fall into two classes, negatively selected ($\omega < 1$) or neutral ($\omega = 1$). In all LRTs log-likelihood differences were compared to the χ^2 distribution for a critical value $\alpha = 0.05$ with three degrees of freedom (df). In cases where Model C had a significantly better fit over the null, only sites with a Bayes empirical Bayes (BEB) posterior probability > 0.80 were considered as significant.

We first applied the clade model C to test for divergent selection between Cetancodonta and the rest of the tree (Clade I in Figure 1). Among 6,894 loci tested, we identified 3,180 for which model C had a better overall fit when compared to the null model prior to any correction for multiple testing (see Additional file 2). Likewise, we also applied model C to each of the Cetacea, Mysticeti and Odontoceti clades (Clades II, III and IV, respectively in Figure 1) and found divergent selection pressures acting on between 4,402 and 5,635 CDSs. Overall, between 1,132 and 2,123 genes had sites with a dN/dS or ω>1 in each of the four clades of interest (see Additional file 2). After filtering for potential false positives (see Material and Methods and Additional file 2), we report 2,169 coding gene sequences under divergent selection in Cetancodonta using Model C. Of these genes, 392 show positively selected sites (PSSs), for which ω>1 in the foreground clade (see Additional files 2-3). We also recovered ~3,600 genes showing evidence of divergent selection acting only in cetaceans, among which 487 to 732 genes exhibited sites with ω>1 on the ancestral cetacean, ancestral odontocete, or ancestral mysticete branches (see Additional files 2-3).

GC content estimation. To control for CpG islands, also known to inflate inferences of natural selection in mammalian genome data, we used the program nhPhyml [12, 13] to infer the GC content at the third codon position (GC3) at each node of the laurasiatherian tree for every CDS dataset (topology was fixed, alpha parameter estimated with four distinct categories and GC equilibrium optimized). We next calculated the shift in GC3 along the five branches (ΔGC3) tested for positive selection (Figure 1), by subtracting the GC3 content between the nodes delineating the branch (as in [14]). To test whether the GC content may have affected our selection results, we calculated the Pearson correlation coefficient between ΔGC3 and the *P* value obtained by the LRTs for a given branch. We found no significant

correlation between strength of selection and shift in GC, with the exception of a slight negative correlation detected for the ancestral branch of Odontoceti (P = 0.0159). In this case the correlation coefficient was very low (r = -0.0251), indicating that GC content is unlikely to have biased our selection analyses (see Additional file 2).

Gene ontology (GO) enrichment analysis. To investigate whether genes showing evidence of molecular adaptation shared similar functions, we used the topGO package [15] to perform an enrichment analysis for GO terms. Gene annotations for the three major GO domains - cellular component (CC), biological process (BP) and molecular function (MF)- were retrieved using Ensembl BioMart [3]. We asked whether genes with PSSs shared specific functions and performed a series of gene ontology (GO) enrichment analyses of genes showing evidence for molecular adaptations in the ancestral branches of Cetacea (pooling branches ii, iii, iv), Cetancodonta and *H. amphibius*. We used two statistics to look for overrepresented terms: (1) the parametric Fisher's exact test and (2) the non-parametric Kolmogorov-Smirnov or else widely known as gene set enrichment analysis (GSEA). In Fisher's test, we set the significance threshold at genes with $Q \leq 0.10$. Both tests were performed following the classic approach, where significance for enrichment was calculated independently for each GO term, as well as using the *elim* and *weight01* algorithms introduced by Alexa et al. 2006 and implemented in the topGO package. Both *elim* and *weight* algorithms were shown to improve the explanatory power of GO group scoring, by eliminating local dependencies between GO terms in the GO graph structure [15].

Using the classic Fisher's exact test to look for overrepresentation of gene sets exhibiting bursts of selection ($Q \leq 0.10$) in cetaceans (pooling branches ii, iii and iv of Figure 1), we detected significant enrichment in 219 GO categories (145 BP, 28 CC, 46 MF; see Additional files 4-5). Among these, the majority of terms was associated with immunity, *e.g.* genes involved in T cell activation (GO:0002286), T cell homeostasis (GO:0043029) or T cell apoptotic process (GO:0070231), or genes that were responsible for interferon and interleukin production (e.g. GO:0032607 and GO:0032613, respectively; see Additional files 4-5). Many GO terms were also linked to the nervous system (GO:0048484, GO:0048485) and/or brain development (e.g. GO:0021549, GO:0021575, GO:0021587, GO:0021695, GO:0021696, GO:0048854) in line with previous findings [16-18]. Finally, we found evidence for enrichment in genes involved in olfaction (GO:0021772, GO:0021988) and oxidation-reduction (redox) reaction (e.g., GO:0016701, GO:0016702).

With respect to the last common ancestor of Cetancodonta, we found 35 significantly enriched GO terms for genes showing evidence of positive selection in Cetancondonta (branch i in Figure 1; 8 terms for BP, 25 for CC and 2 MF; see Additional files 4-5). Of these, seven were related to the protein actin that forms the cytoskeleton (GO:0070252, GO:0030048, GO:0030029, GO:0032432, GO:0005884, GO:0015629, GO:0003779) and others were linked to muscle regulation and the sarcomere (GO:0006937, GO:0090257, GO:0006936, GO:0030017; see Additional file 4-5). GO analysis of the one gene found under positive selection in the hippo branch after FDR correction under the model MA (branch ii in Figure 1, see Additional file 4) recovered the term "vesicle", with roles in vesicle coating (GO:0048208, GO:0048199, GO:0006901) and vesicle targeting (GO:0048207, GO:0048199, GO:0006903). Additional GO terms detected in the hippo were associated with immunity, *e.g.* "antigen processing and presentation via MHC II" (GO:0002495, GO:0002474). Note again our GO results are reflective of the very low number of positively selected genes retained after our filtering steps for false positives. Therefore, we cannot exclude the possibility that a wider range of gene

sets have undergone Darwinian selection in the last common ancestor of hippos and whales.

Next we employed a relaxed approach for identifying functional enrichment associated with increased ω in the foreground branch. Applying Kolmogorov-Smirnov (KS) test coupled with the *elim* method of the TopGO package [15], we ranked all genes based on a score drawn by the goodness-of-fit of the MA selection model (*Q*) and looked for outlier GO sets, as compared to a null distribution GO scores of all loci. This analysis allowed us to detect over- or underrepresented genes in 509 GO categories in Cetacea, 218 terms in Cetancodonta and 213 in *H. amphibius* branches within BP (see Additional file 4). We also found enrichment in 97 and 141 GO terms in all cetaceans within CC and MF domains, respectively. Finally, 44 and 49 GO groups were significantly enriched in hippo and Cetancodonta based on CC domain, whereas 46 in MF in total. A more detailed presentation of all GO terms for each branch and method is provided in Additional file 5. In cetaceans, the majority of GO terms obtained via the KS-elim method based on selection screens seemed to be related to immune response (e.g., GO:0002204, GO:0002208, GO:0002285) and metabolic process (e.g., GO:0006475, GO:0006520, GO:0006541). Analysis of cetacean gene selection results recovered enrichment also in categories related to lipid metabolism (e.g., GO:0042632, GO:0019217, GO:0016126, GO:0019433), blood clotting or platelet formation (e.g.,GO:0007596, GO:0050817), muscle, heart and brain development (e.g., GO:0051145, GO:0003205, GO:0030901), response to stress (e.g.GO:0006950, GO:0033554), hypoxia (GO:0036294) and visual perception (GO:0007601), none of which was obtained by gene set enrichment analysis of selection screen at the hippo branch or at the common ancestor of hippos and whales. Some of these GO terms however may include gene clusters for which we had not sampled the hippo sequences.

Network analysis of protein-protein interactions. GO terms were grouped into functional categories, using associated key terms as follows: the circulatory system (*heart, cardiac, blood, vessel, circulatory, cardio*, coagula*, wound, angio*, vasocon*, vasodil*, lymphocyte, leukocyte, hematopoietic, platelet* and *vascular*); nervous system (*brain, neuro*, nerv*, axon, synap** and *glial*); fluid regulation (*fluid, storage, water* and *urine*); response to hypoxia (*hypoxia*, *break, repair, damage, oxygen* and *oxidative stress*); kidneys (*renal*, *kidney* and *urogenital*); lipids (*lipid, storage* and *cholesterol*); lungs (*lung* and *respiratory*); muscles (*muscle*, *myofibril* and *muscular*); sensory perception (*sensory, visual, vision, eye, perception, cochlea, phototransduction, light, sound* and *retina*) and the cell cycle (*cell cycle, phase, aging* and *telomer**).

References:

1 Grabherr, M. G., Haas, B. J., Yassour, M., Levin, J. Z., Thompson, D. A., Amit, I., Adiconis, X., Fan, L., Raychowdhury, R., Zeng, Q.*, et al.* 2011 Full-length transcriptome assembly from RNA-Seq data without a reference genome. *Nat Biotechnol*. **29**, 644-652. (10.1038/nbt.1883)
2 Holt, C., Yandell, M. 2011 MAKER2: an annotation pipeline and genome-database management tool for second-generation genome projects. *BMC bioinform*. **12**, 491. (10.1186/1471-2105-12-491)
3 Kinsella, R. J., Kahari, A., Haider, S., Zamora, J., Proctor, G., Spudich, G., Almeida-King, J., Staines, D., Derwent, P., Kerhornou, A.*, et al.* 2011 Ensembl BioMarts: a hub for data retrieval across taxonomic space. *Database : j biol datab cur*. **2011**, bar030. (10.1093/database/bar030)
4 Yim, H. S., Cho, Y. S., Guang, X. M., Kang, S. G., Jeong, J. Y., Cha, S. S., Oh, H. M., Lee, J. H., Yang, E. C., Kwon, K. K.*, et al.* 2014 Minke whale genome and aquatic adaptation in cetaceans. *Nat Genet*. **46**, 88-+. (Doi 10.1038/Ng.2835)

5 Loytynoja, A., Goldman, N. 2005 An algorithm for progressive multiple alignment of sequences with insertions. *PNAS*. **102**, 10557-10562. (10.1073/pnas.0409137102)
6 Penn, O., Privman, E., Ashkenazy, H., Landan, G., Graur, D., Pupko, T. 2010 GUIDANCE: a web server for assessing alignment confidence scores. *Nucl acid res*. **38**, W23-28. (10.1093/nar/gkq443)
7 Yandell, M., Ence, D. 2012 A beginner's guide to eukaryotic genome annotation. *Nat Rev Genet*. **13**, 329-342. (10.1038/nrg3174)
8 Yang, Z. H. 2007 PAML 4: Phylogenetic analysis by maximum likelihood. *Mol Biol Evol*. **24**, 1586-1591. (Doi 10.1093/Molbev/Msm088)
9 Zhang, J. Z., Nielsen, R., Yang, Z. H. 2005 Evaluation of an improved branch-site likelihood method for detecting positive selection at the molecular level. *Mol Biol Evol*. **22**, 2472-2479. (Doi 10.1093/Molbev/Msi237)
10 McGowen, M. R., Spaulding, M., Gatesy, J. 2009 Divergence date estimation and a comprehensive molecular tree of extant cetaceans. *Mol phyl evol*. **53**, 891-906. (Doi 10.1016/J.Ympev.2009.08.018)
11 Tsagkogeorga, G., Parker, J., Stupka, E., Cotton, J. A., Rossiter, S. J. 2013 Phylogenomic analyses elucidate the evolutionary relationships of bats. *Curr Biol*. **23**, 2262-2267. (10.1016/j.cub.2013.09.014)
12 Boussau, B., Gouy, M. 2006 Efficient likelihood computations with nonreversible models of evolution. *Syst Biol*. **55**, 756-768. (Doi 10.1080/10635150600975218)
13 Galtier, N., Gouy, M. 1998 Inferring pattern and process: Maximum-likelihood implementation of a nonhomogeneous model of DNA sequence evolution for phylogenetic analysis. *Mol Biol Evol*. **15**, 871-879.
14 Roux, J., Privman, E., Moretti, S., Daub, J. T., Robinson-Rechavi, M., Keller, L. 2014 Patterns of positive selection in seven ant genomes. *Mol Biol Evol*. **31**, 1661-1685. (Doi 10.1093/Molbev/Msu141)
15 Alexa, A., Rahnenfuhrer, J., Lengauer, T. 2006 Improved scoring of functional groups from gene expression data by decorrelating GO graph structure. *Bioinform*. **22**, 1600-1607. (Doi 10.1093/Bioinformatics/Btl140)
16 McGowen, M. R., Grossman, L. I., Wildman, D. E. 2012 Dolphin genome provides evidence for adaptive evolution of nervous system genes and a molecular rate slowdown. *P R Soc B*. **279**, 3643-3651. (Doi 10.1098/Rspb.2012.0869)
17 Nery, M. F., Gonzalez, D. J., Opazo, J. C. 2013 How to make a dolphin: molecular signature of positive selection in cetacean genome. *PloS one*. **8**, (ARTN e65491 DOI 10.1371/journal.pone.0065491)
18 Sun, Y. B., Zhou, W. P., Liu, H. Q., Irwin, D. M., Shen, Y. Y., Zhang, Y. P. 2013 Genome-wide scans for candidate genes in the aquatic adaptation of dolphins. *Gen Biol Evol*. **5**, 130-139. (Doi 10.1093/Gbe/Evs123)

Table S1. RNA extraction QC, RNA-Seq and assembly statistics

	H. amphibius	*H. amphibius*	*H. amphibius*	*M. novaeangliae*
Concentration (ng/μL)	1,026	189	1,797	4,014
Volume (μL)	25	25	35	30
Total Mass (μg)	25.65	4.73	62.9	120.42
RIN	2.4	2.7	3	6
28S/18S	0.7	0	0	0
Total # reads	19,332,863	20,818,962	20,318,928	19,402,616
Total (bp)	3,479,915,340	3,747,413,160	3,657,407,040	3,492,470,880
Q20 %	97.97%	97.95%	97.88%	98.24%
N %	0%	0%	0%	0.01%
GC %	47.49%	45.54%	47.13%	45.89%
Total # assembled transcripts			154,931	290,984
Contig N50 (bp)			720	573

Table S2. Ortholog identification

Species	Data type; Annotation	Reciprocal blast *vs* dolphin *T. truncatus* (Σg =16,549*) ≥50% coverage	(complete)	Reciprocal blast *vs* human *H. sapiens* (Σg =23,658*) ≥50% coverage	(complete)	#Total candidate orthologs
HIPPOPOTAMIDAE						
Hippopotamus *Hippopotamus amphibius*	RNA-seq; *de novo* assembly	7,394	(2,707)	7,921	(2,719)	**10,834**
CETACEA						
MYSTICETI						
Humpback whale *Megaptera novaeangliae*	RNA-seq; *de novo* assembly	7,831	(2,479)	8,314	(2,396)	**10,875**
Minke whale *Balaenoptera acutorostrata*	genomic; gene prediction	12,940	(7,747)	14,067	(8,344)	**14,234**
Fin whale *Balaenoptera physalus*	genomic; SNP calling	12,908	(7,647)	14,016	(8,219)	**14,056**
ODONTOCETI						
Yangtze river dolphin *Lipotes vexillifer*	genomic; gene prediction	11,543	(4,787)	12,865	(5,367)	**13,635**
Finless porpoise *Neophocaena phocaenoides*	genomic; SNP calling	16,047	(13,064)	15,082	(9,881)	**14,868**
Killer whale *Orcinus orca*	genomic; gene prediction	9,323	(7,281)	9,476	(7,981)	**9,267**
Indo-Pacific humpback dolphin *Sousa chinensis*	RNA-seq; *de novo* assembly	6,249	(2,409)	6,667	(2,425)	**9,360**
Sperm whale *Physeter macrocephalus*	RNA-seq; *de novo* assembly	9,493	(3,461)	10,114	(3,609)	**11,782**

*translated protein-coding genes

Table S3. Genome-wide analysis for bursts of divergent selection

	Initial screen for selection			Filtering	# Genes showing evidence of natural selection			
	# Total Datasets		*p-val* < 0.05	# Datasets Med PSSs ≤ 10	*p-val* < 0.05		FDR *q-val* < 0.10	
Clade model C		$\omega \neq \omega_0$	$\omega \neq \omega_0$, $\omega > 1$		$\omega \neq \omega_0$	$\omega \neq \omega_0$, $\omega > 1$	$\omega \neq \omega_0$	$\omega \neq \omega_0$, $\omega > 1$
Cetancodonta clade	6,894	3,180	1,132	462	2,235	**412**	2,169	**392**
Cetacea clade	11,992	5,635	2,123	901	3,967	**761**	3,872	**732**
Mysticeti clade	11,992	4,402	1,252	519	3,577	**539**	3,386	**487**
Odontoceti clade	11,992	4,614	1.436	469	3,762	**732**	3,623	**701**
				# Genes excluded = 2,063				

Table S4. Pearson correlation test between MA model fit (LRT *p-values*) and ΔGC3 at the third codon position of the branch

Branch tested	Correlation coefficient (*r*)	*p*-value
Ancestral Cetancodonta (hippo + cetaceans)	-0.0081	0.5537
Ancestral Cetacea	-0.0059	0.5683
Ancestral Mysticeti	-0.0134	0.1997
Ancestral Odontoceti	**-0.0251**	**0.0159**
H. amphibius terminal branch	0.0054	0.6944

Table S7A. TopGO enrichment results for positively selected genes based on p-values from the branch-site model.

Branch tested	Biological Process (BP)		Cellular Component (CC)		Molecular Function (MF)	
	Fisher Classic	KS Elim	Fisher Classic	KS Elim	Fisher Classic	KS Elim
Ancestral Cetancodonta	8	218	25	49	2	46
H. amphibius	43	215	8	44	1	46
Ancestral Cetacea	44	389	12	73	20	118
Ansestral Mysticeti	38	401	13	73	10	111
Ancestral Odontoceti	80	417	3	82	19	115
Union:	145	**509**	28	**97**	46	**141**

Table S7B. GO terms found enriched in positively selected genes in hippo and whales, based on Fisher's exact test.

GO ID	GO domain	Term	(iv) Cetancodonta
GO:0070252	BP	actin-mediated cell contraction	0.0032
GO:0030048	BP	actin filament-based movement	0.0047
GO:0006937	BP	regulation of muscle contraction	0.0074
GO:0090257	BP	regulation of muscle system process	0.0096
GO:0006936	BP	muscle contraction	0.0179
GO:0044057	BP	regulation of system process	0.0189
GO:0003012	BP	muscle system process	0.0219
GO:0030029	BP	actin filament-based process	0.0385
GO:0002102	CC	podosome	0.0020
GO:0001725	CC	stress fiber	0.0030
GO:0030863	CC	cortical cytoskeleton	0.0030
GO:0032154	CC	cleavage furrow	0.0030
GO:0032432	CC	actin filament bundle	0.0030
GO:0005884	CC	actin filament	0.0034
GO:0032153	CC	cell division site	0.0034
GO:0032155	CC	cell division site part	0.0034
GO:0042641	CC	actomyosin	0.0034
GO:0005604	CC	basement membrane	0.0059
GO:0030426	CC	growth cone	0.0065
GO:0044448	CC	cell cortex part	0.0065
GO:0030427	CC	site of polarized growth	0.0069
GO:0044420	CC	extracellular matrix part	0.0097
GO:0030017	CC	sarcomere	0.0105
GO:0044449	CC	contractile fiber part	0.0115
GO:0030016	CC	myofibril	0.0129
GO:0043292	CC	contractile fiber	0.0133
GO:0005938	CC	cell cortex	0.0143
GO:0005578	CC	proteinaceous extracellular matrix	0.0168
GO:0031012	CC	extracellular matrix	0.0198
GO:0015629	CC	actin cytoskeleton	0.0244
GO:0044463	CC	cell projection part	0.0347
GO:0043005	CC	neuron projection	0.0408
GO:0097458	CC	neuron part	0.0485
GO:0003779	MF	actin binding	0.0230

GO:0008092	MF	cytoskeletal protein binding	0.0460

Table S7C. GO terms enriched in positively selected genes in the hippo, based on Fisher's exact test.

GO ID	GO domain	Term	(v) Hippo
GO:0048207	BP	vesicle targeting, rough ER to cis-Golgi	0.0051
GO:0048208	BP	COPII vesicle coating	0.0051
GO:0090114	BP	COPII-coated vesicle budding	0.0051
GO:0048199	BP	vesicle targeting, to, from or within Golgi	0.0090
GO:0006901	BP	vesicle coating	0.0098
GO:1902591	BP	single-organism membrane budding	0.0098
GO:0006903	BP	vesicle targeting	0.0111
GO:0006900	BP	membrane budding	0.0119
GO:0051592	BP	response to calcium ion	0.0136
GO:0042787	BP	protein ubiquitination involved in ubiquitin-dependent protein catabolic process	0.0141
GO:0006888	BP	ER to Golgi vesicle-mediated transport	0.0153
GO:0002495	BP	antigen processing and presentation of peptide antigen via MHC class II	0.0158
GO:0002504	BP	antigen processing and presentation of peptide or polysaccharide antigen via MHC class II	0.0158
GO:0019886	BP	antigen processing and presentation of exogenous peptide antigen via MHC class II	0.0158
GO:0006987	BP	activation of signaling protein activity involved in unfolded protein response	0.0174
GO:0032075	BP	positive regulation of nuclease activity	0.0179
GO:0032069	BP	regulation of nuclease activity	0.0183
GO:0002474	BP	antigen processing and presentation of peptide antigen via MHC class I	0.0217
GO:0030968	BP	endoplasmic reticulum unfolded protein response	0.0217
GO:0016050	BP	vesicle organization	0.0221
GO:0034620	BP	cellular response to unfolded protein	0.0221
GO:0035967	BP	cellular response to topologically incorrect protein	0.0225
GO:0018279	BP	protein N-linked glycosylation via asparagine	0.0234
GO:0051650	BP	establishment of vesicle localization	0.0234
GO:0018196	BP	peptidyl-asparagine modification	0.0238
GO:0051648	BP	vesicle localization	0.0242
GO:0006984	BP	ER-nucleus signaling pathway	0.0246
GO:0006487	BP	protein N-linked glycosylation	0.0255
GO:0034976	BP	response to endoplasmic reticulum stress	0.0301
GO:0002478	BP	antigen processing and presentation of exogenous peptide antigen	0.0318
GO:0006986	BP	response to unfolded protein	0.0318
GO:0019884	BP	antigen processing and presentation of exogenous antigen	0.0322
GO:0035966	BP	response to topologically incorrect protein	0.0322
GO:0010038	BP	response to metal ion	0.0335
GO:0048002	BP	antigen processing and presentation of peptide antigen	0.0335
GO:0051656	BP	establishment of organelle localization	0.0343
GO:0019882	BP	antigen processing and presentation	0.0347
GO:0007283	BP	spermatogenesis	0.0402
GO:0043687	BP	post-translational protein modification	0.0402
GO:0048232	BP	male gamete generation	0.0406
GO:0051640	BP	organelle localization	0.0423

GO:0048193	BP	Golgi vesicle transport	0.0427
GO:0010035	BP	response to inorganic substance	0.0469
GO:0012507	CC	ER to Golgi transport vesicle membrane	0.0061
GO:0030134	CC	ER to Golgi transport vesicle	0.0091
GO:0030120	CC	vesicle coat	0.0133
GO:0030658	CC	transport vesicle membrane	0.0133
GO:0030117	CC	membrane coat	0.0242
GO:0048475	CC	coated membrane	0.0242
GO:0030662	CC	coated vesicle membrane	0.0296
GO:0030133	CC	transport vesicle	0.0313
GO:0048306	MF	calcium-dependent protein binding	0.0062

Table S7D. GO terms enriched for positively selected genes in cetaceans, based on Fisher's exact test.

GO ID	GO domain	Term	(i) Cetacea	(ii) Mysticeti	(iii) Odontoceti
GO:0000086	BP	G2/M transition of mitotic cell cycle	0.0029	0.0216	0.0103
GO:0000226	BP	microtubule cytoskeleton organization	0.0139	NS	NS
GO:0001539	BP	ciliary or bacterial-type flagellar motility	NS	NS	0.0260
GO:0001755	BP	neural crest cell migration	NS	0.0486	NS
GO:0001763	BP	morphogenesis of a branching structure	NS	0.0493	NS
GO:0001816	BP	cytokine production	NS	NS	0.0085
GO:0001817	BP	regulation of cytokine production	NS	NS	0.0068
GO:0001818	BP	negative regulation of cytokine production	NS	NS	0.0059
GO:0001906	BP	cell killing	0.0238	NS	NS
GO:0001909	BP	leukocyte mediated cytotoxicity	0.0209	NS	NS
GO:0002228	BP	natural killer cell mediated immunity	0.0130	NS	NS
GO:0002252	BP	immune effector process	0.0229	NS	NS
GO:0002260	BP	lymphocyte homeostasis	NS	NS	0.0497
GO:0002286	BP	T cell activation involved in immune response	NS	NS	0.0443
GO:0002287	BP	alpha-beta T cell activation involved in immune response	NS	NS	0.0352
GO:0002292	BP	T cell differentiation involved in immune response	NS	NS	0.0352
GO:0002293	BP	alpha-beta T cell differentiation involved in immune response	NS	NS	0.0352
GO:0002294	BP	CD4-positive, alpha-beta T cell differentiation involved in immune response	NS	NS	0.0334
GO:0002367	BP	cytokine production involved in immune response	NS	NS	0.0443
GO:0002718	BP	regulation of cytokine production involved in immune response	NS	NS	0.0388
GO:0002753	BP	cytoplasmic pattern recognition receptor signaling pathway	NS	NS	0.0443
GO:0002831	BP	regulation of response to biotic stimulus	0.0471	NS	NS
GO:0002833	BP	positive regulation of response to biotic stimulus	NS	0.0273	NS
GO:0006457	BP	protein folding	0.0057	NS	NS
GO:0006458	BP	'*de novo*' protein folding	0.0307	NS	NS
GO:0006928	BP	cellular component movement	0.0430	NS	NS
GO:0007017	BP	microtubule-based process	0.0289	NS	NS
GO:0007051	BP	spindle organization	0.0013	NS	NS

GO:0007156	BP	homophilic cell adhesion	NS	NS	0.0060
GO:0007157	BP	heterophilic cell-cell adhesion	NS	NS	0.0260
GO:0007260	BP	tyrosine phosphorylation of STAT protein	NS	NS	0.0334
GO:0007566	BP	embryo implantation	NS	NS	0.0443
GO:0009072	BP	aromatic amino acid family metabolic process	NS	0.0459	NS
GO:0009074	BP	aromatic amino acid family catabolic process	NS	0.0326	NS
GO:0009890	BP	negative regulation of biosynthetic process	NS	NS	0.0282
GO:0010558	BP	negative regulation of macromolecule biosynthetic process	NS	NS	0.0237
GO:0010559	BP	regulation of glycoprotein biosynthetic process	NS	NS	0.0315
GO:0016337	BP	cell-cell adhesion	NS	NS	0.0094
GO:0016339	BP	calcium-dependent cell-cell adhesion	NS	NS	0.0279
GO:0016925	BP	protein sumoylation	NS	NS	0.0334
GO:0021549	BP	cerebellum development	0.0491	NS	NS
GO:0021575	BP	hindbrain morphogenesis	0.0268	NS	0.0497
GO:0021587	BP	cerebellum morphogenesis	0.0238	NS	0.0443
GO:0021695	BP	cerebellar cortex development	0.0258	NS	0.0479
GO:0021696	BP	cerebellar cortex morphogenesis	0.0179	0.0486	0.0334
GO:0021772	BP	olfactory bulb development	0.0159	0.0433	0.0297
GO:0021988	BP	olfactory lobe development	0.0169	0.0459	0.0315
GO:0030030	BP	cell projection organization	NS	0.0249	NS
GO:0030031	BP	cell projection assembly	NS	NS	0.0266
GO:0030318	BP	melanocyte differentiation	NS	0.0433	NS
GO:0031327	BP	negative regulation of cellular biosynthetic process	NS	NS	0.0271
GO:0032091	BP	negative regulation of protein binding	0.0199	NS	NS
GO:0032319	BP	regulation of Rho GTPase activity	NS	0.0183	NS
GO:0032320	BP	positive regulation of Ras GTPase activity	NS	0.0231	NS
GO:0032321	BP	positive regulation of Rho GTPase activity	NS	0.0095	NS
GO:0032480	BP	negative regulation of type I interferon production	NS	NS	0.0334
GO:0032607	BP	interferon-alpha production	NS	NS	0.0187
GO:0032608	BP	interferon-beta production	NS	NS	0.0425
GO:0032613	BP	interleukin-10 production	NS	NS	0.0242
GO:0032615	BP	interleukin-12 production	NS	NS	0.0461
GO:0032635	BP	interleukin-6 production	0.0336	NS	NS
GO:0032647	BP	regulation of interferon-alpha production	NS	NS	0.0187
GO:0032648	BP	regulation of interferon-beta production	NS	NS	0.0406
GO:0032653	BP	regulation of interleukin-10 production	NS	NS	0.0224
GO:0032655	BP	regulation of interleukin-12 production	NS	NS	0.0443
GO:0032675	BP	regulation of interleukin-6 production	0.0336	NS	NS
GO:0032728	BP	positive regulation of interferon-beta production	NS	NS	0.0279
GO:0032956	BP	regulation of actin cytoskeleton organization	NS	0.0414	NS
GO:0032990	BP	cell part morphogenesis	NS	0.0348	NS
GO:0034381	BP	plasma lipoprotein particle clearance	0.0179	NS	NS
GO:0035023	BP	regulation of Rho protein signal transduction	NS	0.0414	NS

GO:0036230	BP	granulocyte activation	NS	0.0326	NS
GO:0039528	BP	cytoplasmic pattern recognition receptor signaling pathway in response to virus	NS	0.0273	0.0187
GO:0042035	BP	regulation of cytokine biosynthetic process	0.0462	NS	NS
GO:0042036	BP	negative regulation of cytokine biosynthetic process	0.0169	NS	0.0315
GO:0042058	BP	regulation of epidermal growth factor receptor signaling pathway	0.0394	NS	NS
GO:0042059	BP	negative regulation of epidermal growth factor receptor signaling pathway	0.0248	NS	NS
GO:0042088	BP	T-helper 1 type immune response	NS	NS	0.0242
GO:0042089	BP	cytokine biosynthetic process	0.0471	NS	NS
GO:0042093	BP	T-helper cell differentiation	NS	NS	0.0334
GO:0042107	BP	cytokine metabolic process	0.0481	NS	NS
GO:0042119	BP	neutrophil activation	NS	0.0326	NS
GO:0042267	BP	natural killer cell mediated cytotoxicity	0.0130	NS	NS
GO:0043029	BP	T cell homeostasis	NS	NS	0.0297
GO:0043367	BP	CD4-positive, alpha-beta T cell differentiation	NS	NS	0.0479
GO:0043370	BP	regulation of CD4-positive, alpha-beta T cell differentiation	NS	NS	0.0315
GO:0043547	BP	positive regulation of GTPase activity	NS	0.0446	NS
GO:0043900	BP	regulation of multi-organism process	NS	0.0300	NS
GO:0043902	BP	positive regulation of multi-organism process	NS	0.0059	NS
GO:0044770	BP	cell cycle phase transition	0.0258	NS	NS
GO:0044772	BP	mitotic cell cycle phase transition	0.0256	NS	NS
GO:0044839	BP	cell cycle G2/M phase transition	0.0029	0.0216	0.0103
GO:0045581	BP	negative regulation of T cell differentiation	NS	NS	0.0297
GO:0045620	BP	negative regulation of lymphocyte differentiation	NS	NS	0.0352
GO:0045622	BP	regulation of T-helper cell differentiation	NS	NS	0.0242
GO:0045661	BP	regulation of myoblast differentiation	NS	NS	0.0315
GO:0046040	BP	IMP metabolic process	NS	0.0300	NS
GO:0046636	BP	negative regulation of alpha-beta T cell activation	NS	NS	0.0187
GO:0046637	BP	regulation of alpha-beta T cell differentiation	NS	NS	0.0479
GO:0048484	BP	enteric nervous system development	NS	0.0273	NS
GO:0048485	BP	sympathetic nervous system development	NS	0.0326	NS
GO:0048741	BP	skeletal muscle fiber development	NS	NS	0.0497
GO:0048742	BP	regulation of skeletal muscle fiber development	NS	NS	0.0388
GO:0048747	BP	muscle fiber development	NS	0.0046	NS
GO:0048846	BP	axon extension involved in axon guidance	NS	0.0353	NS
GO:0048854	BP	brain morphogenesis	0.0209	NS	0.0370
GO:0048858	BP	cell projection morphogenesis	NS	0.0324	NS
GO:0050688	BP	regulation of defense response to virus	0.0375	NS	NS
GO:0050690	BP	regulation of defense response to virus by virus	0.0159	NS	NS
GO:0051084	BP	*'de novo'* posttranslational protein folding	0.0277	NS	NS
GO:0051100	BP	negative regulation of binding	0.0404	NS	NS
GO:0051146	BP	striated muscle cell differentiation	NS	0.0480	NS

GO:0051148	BP	negative regulation of muscle cell differentiation	NS	NS	0.0425
GO:0051225	BP	spindle assembly	0.0297	NS	NS
GO:0051241	BP	negative regulation of multicellular organismal process	NS	NS	0.0457
GO:0051489	BP	regulation of filopodium assembly	NS	NS	0.0370
GO:0051491	BP	positive regulation of filopodium assembly	NS	NS	0.0279
GO:0055001	BP	muscle cell development	NS	0.0207	NS
GO:0055002	BP	striated muscle cell development	NS	0.0183	NS
GO:0060008	BP	Sertoli cell differentiation	0.0110	NS	NS
GO:0060271	BP	cilium morphogenesis	NS	0.0197	NS
GO:0060396	BP	growth hormone receptor signaling pathway	NS	NS	0.0315
GO:0060397	BP	JAK-STAT cascade involved in growth hormone signaling pathway	NS	NS	0.0242
GO:0060416	BP	response to growth hormone	NS	NS	0.0388
GO:0061138	BP	morphogenesis of a branching epithelium	NS	0.0459	NS
GO:0070227	BP	lymphocyte apoptotic process	NS	NS	0.0479
GO:0070228	BP	regulation of lymphocyte apoptotic process	NS	NS	0.0406
GO:0070229	BP	negative regulation of lymphocyte apoptotic process	NS	NS	0.0242
GO:0070231	BP	T cell apoptotic process	NS	NS	0.0370
GO:0070232	BP	regulation of T cell apoptotic process	NS	NS	0.0297
GO:0070242	BP	thymocyte apoptotic process	NS	NS	0.0187
GO:0070670	BP	response to interleukin-4	NS	NS	0.0297
GO:0070925	BP	organelle assembly	0.0120	NS	NS
GO:0071353	BP	cellular response to interleukin-4	NS	NS	0.0297
GO:0071378	BP	cellular response to growth hormone stimulus	NS	NS	0.0315
GO:0071526	BP	semaphorin-plexin signaling pathway	NS	0.0300	NS
GO:0097006	BP	regulation of plasma lipoprotein particle levels	0.0326	NS	NS
GO:0098586	BP	cellular response to virus	NS	0.0300	0.0205
GO:1901184	BP	regulation of ERBB signaling pathway	0.0404	NS	NS
GO:1901185	BP	negative regulation of ERBB signaling pathway	0.0258	NS	NS
GO:1902284	BP	neuron projection extension involved in neuron projection guidance	NS	0.0353	NS
GO:2000107	BP	negative regulation of leukocyte apoptotic process	NS	NS	0.0334
GO:2000108	BP	positive regulation of leukocyte apoptotic process	NS	NS	0.0242
GO:2000514	BP	regulation of CD4-positive, alpha-beta T cell activation	NS	NS	0.0334
GO:2001241	BP	positive regulation of extrinsic apoptotic signaling pathway in absence of ligand	NS	NS	0.0205
GO:0000313	CC	organellar ribosome	NS	0.0367	NS
GO:0005576	CC	extracellular region	0.0390	NS	NS
GO:0005761	CC	mitochondrial ribosome	NS	0.0367	NS
GO:0005776	CC	autophagic vacuole	NS	0.0439	NS
GO:0005791	CC	rough endoplasmic reticulum	0.0280	NS	NS
GO:0005814	CC	centriole	0.0350	NS	NS
GO:0005868	CC	cytoplasmic dynein complex	NS	NS	0.0260
GO:0005874	CC	microtubule	0.0170	NS	NS
GO:0005905	CC	coated pit	0.0370	NS	NS

GO:0008023	CC	transcription elongation factor complex	NS	0.0463	NS
GO:0030016	CC	myofibril	NS	0.0212	NS
GO:0030017	CC	sarcomere	NS	0.0147	NS
GO:0030018	CC	Z disc	NS	0.0052	NS
GO:0030118	CC	clathrin coat	0.0310	NS	NS
GO:0030119	CC	AP-type membrane coat adaptor complex	0.0270	NS	NS
GO:0030120	CC	vesicle coat	0.0290	NS	NS
GO:0030125	CC	clathrin vesicle coat	0.0120	NS	NS
GO:0030131	CC	clathrin adaptor complex	0.0230	NS	NS
GO:0030286	CC	dynein complex	NS	NS	0.0350
GO:0030669	CC	clathrin-coated endocytic vesicle membrane	0.0100	NS	NS
GO:0031674	CC	I band	NS	0.0075	NS
GO:0031941	CC	filamentous actin	NS	0.0343	NS
GO:0043034	CC	costamere	NS	0.0295	NS
GO:0043198	CC	dendritic shaft	NS	0.0463	NS
GO:0043292	CC	contractile fiber	NS	0.0234	NS
GO:0044447	CC	axoneme part	NS	NS	0.0280
GO:0044449	CC	contractile fiber part	NS	0.0178	NS
GO:0045334	CC	clathrin-coated endocytic vesicle	0.0130	NS	NS
GO:0000166	MF	nucleotide binding	NS	NS	0.0340
GO:0001871	MF	pattern binding	0.0100	NS	0.0210
GO:0001882	MF	nucleoside binding	NS	NS	0.0440
GO:0001883	MF	purine nucleoside binding	NS	NS	0.0430
GO:0003690	MF	double-stranded DNA binding	NS	0.0166	NS
GO:0003725	MF	double-stranded RNA binding	0.0288	NS	NS
GO:0004672	MF	protein kinase activity	NS	NS	0.0280
GO:0005044	MF	scavenger receptor activity	0.0223	NS	0.0470
GO:0005085	MF	guanyl-nucleotide exchange factor activity	NS	0.0365	NS
GO:0005088	MF	Ras guanyl-nucleotide exchange factor activity	NS	0.0179	NS
GO:0005089	MF	Rho guanyl-nucleotide exchange factor activity	NS	0.0069	NS
GO:0005200	MF	structural constituent of cytoskeleton	0.0353	NS	NS
GO:0005319	MF	lipid transporter activity	0.0320	NS	NS
GO:0005342	MF	organic acid transmembrane transporter activity	0.0489	NS	NS
GO:0005343	MF	organic acid:sodium symporter activity	0.0182	NS	NS
GO:0005524	MF	ATP binding	NS	NS	0.0200
GO:0008028	MF	monocarboxylic acid transmembrane transporter activity	0.0157	NS	NS
GO:0008565	MF	protein transporter activity	0.0417	NS	NS
GO:0015294	MF	solute:cation symporter activity	0.0409	NS	NS
GO:0015296	MF	anion:cation symporter activity	0.0239	NS	NS
GO:0015370	MF	solute:sodium symporter activity	0.0296	NS	NS
GO:0016701	MF	oxidoreductase activity, acting on single donors with incorporation of molecular oxygen	NS	0.0336	NS
GO:0016702	MF	oxidoreductase activity, acting on single donors with incorporation of molecular oxygen, incorporation of two atoms of oxygen	NS	0.0259	NS

GO:0016773	MF	phosphotransferase activity, alcohol group as acceptor	NS	NS	0.0470
GO:0016805	MF	dipeptidase activity	NS	0.0285	NS
GO:0017076	MF	purine nucleotide binding	NS	NS	0.0470
GO:0030247	MF	polysaccharide binding	0.0100	NS	0.0210
GO:0030506	MF	ankyrin binding	NS	0.0336	NS
GO:0030545	MF	receptor regulator activity	0.0215	NS	NS
GO:0030554	MF	adenyl nucleotide binding	NS	NS	0.0230
GO:0032266	MF	phosphatidylinositol-3-phosphate binding	NS	0.0336	NS
GO:0032549	MF	ribonucleoside binding	NS	NS	0.0430
GO:0032550	MF	purine ribonucleoside binding	NS	NS	0.0430
GO:0032553	MF	ribonucleotide binding	NS	NS	0.0470
GO:0032555	MF	purine ribonucleotide binding	NS	NS	0.0460
GO:0032559	MF	adenyl ribonucleotide binding	NS	NS	0.0220
GO:0035639	MF	purine ribonucleoside triphosphate binding	NS	NS	0.0420
GO:0038024	MF	cargo receptor activity	0.0004	NS	NS
GO:0042287	MF	MHC protein binding	0.0083	NS	NS
GO:0043167	MF	ion binding	NS	NS	0.0330
GO:0043566	MF	structure-specific DNA binding	NS	0.0412	NS
GO:0046943	MF	carboxylic acid transmembrane transporter activity	0.0481	NS	NS
GO:0050750	MF	low-density lipoprotein particle receptor binding	0.0083	NS	NS
GO:0051082	MF	unfolded protein binding	0.0010	NS	NS
GO:0070325	MF	lipoprotein particle receptor binding	0.0108	NS	NS
GO:1901265	MF	nucleoside phosphate binding	NS	NS	0.0340
